# Huaier Polysaccharides Sensitize Anti-PD-L1 Therapy via Promoting Antitumor Immune Response in Triple-Negative Breast Cancer

**DOI:** 10.7150/ijbs.133068

**Published:** 2026-05-18

**Authors:** Lin-xi Zhou, Zi-wei Wu, Yuan Tian, Ke-fei Luo, Qin-wen Pan, Yuan-yin Xi, Peng Tang, Ling-juan Zeng, Ling Zhong, Jun Jiang, Yi Zhang, Lingmi Hou, Hong Zheng, Ming-hao Wang

**Affiliations:** 1 Department of Breast and Thyroid Surgery, Southwest Hospital, Army Medical University, Chongqing, China.; 2 Zhejiang Provincial Key Laboratory of Pancreatic Disease, The First Affiliated Hospital, Zhejiang University School of Medicine, Hangzhou, China.; 3 Research Institute of General Surgery, Jinling Hospital, Medical School of Nanjing University, Nanjing, China.; 4 Department of Breast Surgery, Linyi People's Hospital, Linyi, China.; 5 Department of Breast and Thyroid Surgery/Key Laboratory of Chongqing Health Commission for Minimally Invasive and Precise Diagnosis and Treatment of Breast Cancer, Southwest Hospital, Army Medical University, Chongqing, China.; 6 Department of Breast Surgery, Sichuan Clinical Research Center for Cancer, Sichuan Cancer Hospital & Institute, Sichuan Cancer Center, Affiliated Cancer Hospital of University of Electronic Science and Technology of China, Chengdu, China.; 7 Department of Thoracic Surgery, Xinqiao Hospital, Army Medical University, Chongqing, China.

**Keywords:** triple-negative breast cancer, anti-PD-L1, Huaier, antitumor immunity, autophagy

## Abstract

Triple-negative breast cancer (TNBC) is characterized by its aggressiveness and resistance to conventional therapies. Although immune checkpoint inhibitors (ICIs) have shown promise in cancer treatment, TNBC patients respond suboptimally to ICIs as monotherapy. Our prior clinical research demonstrated that Huaier significantly improved 5-year overall survival and disease-free survival when used as an adjunct to chemotherapy in TNBC patients. However, whether PS-T (the primary component of Huaier granules) enhances the efficacy of ICIs against TNBC and the underlying mechanisms remain unclear. In this study, we found that combining anti-PD-L1 antibodies with PS-T synergistically suppressed tumor growth and cell proliferation in TNBC, accompanied by enhanced antitumor immune responses. Mechanistically, PS-T promoted the autophagic degradation of PD-L1, which in turn facilitated the accumulation and activation of CD45+, CD3+ T, CD4+ T, CD8+ T, and dendritic cells, while reducing immunosuppressive regulatory T cells, ultimately enhancing the efficacy of anti-PD-L1 antibodies.

## Introduction

Among all types of breast cancer, triple-negative breast cancer (TNBC) is the most aggressive, known for its high metastasis rate, recurrence, and a poor prognosis 1. This malignancy lacks estrogen receptor, progesterone receptor, and human epidermal growth factor receptor 2 expression, precluding endocrine and targeted therapies and making chemotherapy the mainstay of treatment 2. Despite this, only about 31% of TNBC patients achieve a pathologic complete response with chemotherapy, and all metastatic TNBC patients eventually develop chemoresistance 3, 4. Consequently, there is an urgent demand for novel treatment strategies to combat TNBC.

In the past decade, immunotherapy with immune checkpoint inhibitors (ICI) has rapidly progressed as a promising therapeutic approach 5. Its efficacy in treating various tumor has been well documented 6, 7. However, TNBC patients exhibit a lower-than-average sensitivity to ICI therapy, with only approximately 20% responding to ICIs 8. This reduced sensitivity may be attributed to the lower prevalence of tumor-infiltrating immune cells in breast cancer compared to other tumors types 9. Studies have suggested that tumor immune infiltrates, T cell function, and tumor mutation burden are closely associated with the responsiveness to immunotherapy 10, 11. These insights imply that increasing the presence of tumor-infiltrating immune cells and boosting T cell cytotoxicity could potentially improve sensitivity to ICI treatment of TNBC patients.

Traditional Chinese Medicine (TCM) has been recognized for its unique benefits in cancer treatment 12, 13. Huaier granules, in particular, have been employed as an adjuvant therapy for various malignancies, showcasing therapeutic efficacy with excellent safety profiles and minimal side effects 14, 15. Our previous clinical study demonstrated that patients with advanced TNBC who received Huaier granules in conjunction with chemotherapy experienced a significant improvement in their 5-year overall survival rate (87.5% vs 67.4%) and disease-free survival rate (81.3% vs 53.8%). In the experimental group, only 13 out of 101 patients experienced disease progression, underscoring the potential of Huaier granules in the management of TNBC 16. The primary active component of Huaier, PS-T (polysaccharides of *Trametes robiniophila Murr*), has demonstrated the ability to induce apoptosis, suppress cell stemness, and impede invasion and metastasis in TNBC 17-19. Recently, it is reported that PS-T may also potentiate antitumor immune response in cholangiocarcinoma and hepatocellular carcinoma 20, 21. PS-T stands out from other sensitizers by not only augmenting treatment sensitivity but also exerting direct antitumor effects, such as inhibiting metastasis and recurrence, while maintaining a low side effect profile 22. Nevertheless, the exact influence of PS-T on the tumor immune microenvironment in TNBC, as well as its potential to sensitize the effects of ICI and the underlying mechanisms, remain to be clarified.

This study was undertaken to evaluate the therapeutic potential of PS-T, when in conjunction with immune checkpoint inhibitors, for the treatment of TNBC, and to delve into its impact and distinct mechanisms on tumor immune cell infiltration. To our knowledge, this represents the first study demonstrating that PS-T triggers autophagy-dependent degradation of PD-L1 and to elucidate the role of PS-T-mediated PD-L1 degradation in the enhancement of anti-tumor immune responses within TNBC. Consequently, this research offers a promising clinical approach against TNBC.

## Materials and Methods

### Chemicals and reagents

Huaier crude extract was provided by Qidong Gaitianli Pharmaceutical Co. Ltd. (Qidong, China). We isolated and purified the polysaccharides (PS-T), as previously reported 18. Phenol-sulfuric acid method with glucose was used to determine the purity of PS-T 23. The autophagy inhibitor 3-methyladenine (3-MA, T1879) and Chloroquine (CQ, T8689), proteasome inhibitor MG132 (T2154), and anti-PD-L1 antibody atezolizumab (Ate, T9902) were purchased from TargetMol (Shanghai, China).

PD-L1 (DF6526), p62 (AF5384), Beclin-1 (AF5128), ATG5 (DF6010) and β-actin (AF7018) antibodies were from Affinity Biosciences (Cincinnati, OH, USA). Anti-PD-L1 (NBP1-43262), LC3 (NB100-2220), CD4 (NBP1-19371), CD8 (NB200-578), FoxP3 (NB100-39002) and Ki67 (NB110-89717) antibodies were from Novus Biologicals (Littleton, CO, USA). Anti-PD-L1 (#13684) and anti-rabbit (#7074) antibodies were from Cell Signaling Technology (Danvers, MA, USA). FITC-conjugated anti-rabbit IgG (E031220) and Cy3-conjugated anti-rat IgG (E031640) were from EarthOx Life Sciences (Millbrae, CA, USA).

The following antibodies for flow cytometry were from BioLegend (San Diego, CA, USA): anti-CD274 (APC, 329708), anti-CD274 (APC, 124312), anti-CD45 (PerCP-CY5.5, 147706), anti-CD45 (APC-Cy7, 109824), anti-CD3 (PE, 100206), anti-CD4 (APC-Cy7, 100414), anti-CD8a (FITC, 100706), anti-granzyme B (PE-Cy7, 396410), anti-IFN-γ (APC, 505810), anti-MHC-II (PerCP-CY5.5, 116416), anti-CD11c (APC, 117310), anti-PD-1 (PE-Cy7, 135216), and anti-LAG-3 (RB705, 756906). Anti-FoxP3 (PerCP-CD5.5, 563902), anti-CD8 (RB545, 569278), and anti-TIM-3 (PE Dazzle 594, 119747) was purchased from BD Biosciences (Pasadena, CA, USA).

### Xenograft experiments

BALB/c mice (5-6 weeks, 18-20 g, female) were provided by Byrness Weil Biotech, Ltd. The Laboratory Animal Welfare and Ethics Committee of the Army Medical University has approved all *in vivo* experiments. Mice were randomly assigned to different groups. 1×10^6^ 4T1 cells were injected into their mammary fat pads. The next day, the mice were separately treated with 100 μL isotonic sodium chloride solution and 3 mg PS-T solution by oral administration every other day. For anti-PD-L1 treatment, 200 μg atezolizumab was intraperitoneally administered once a week, either singly or in combination with PS-T. For mice requiring autophagy inhibitor treatment, 150 μL of 300 μg 3-MA solution was injected intraperitoneally every three days. The drug dosages and administration methods were based on previous literature for optimal consistency 24, 25. From the fifth day, we measured tumor volumes every two days. Mice were euthanized 21 days later, and their tumors and spleens were dissected for subsequent experiments.

### Single-cell RNA-sequencing and analysis

The excised xenograft tumors were from mice at 21 days post-inoculation, and carefully processed into a single-cell suspension. The suspension was then subjected to the 10x Genomics Chromium instrument to generate Gel Bead-in-emulsion (GEMs), with each cell being loaded into a separate GEM for subsequent processing. To ensure comprehensive coverage, we performed sequencing with an average depth of 5,000 reads per cell, which facilitated robust detection of gene expression levels across the individual cells. Following the generation of GEMs, these were transferred to a PCR instrument for reverse transcription, converting the RNA into complementary DNA (cDNA). The cDNA underwent purification, amplification, fragmentation, end-repair, and A-tailing before being ligated with the Read2 sequencing primer. This process resulted in the construction of a cDNA library equipped with P5 and P7 connexons, which was then sequenced in accordance with the Illumina User Guide. The sequencing was carried out in Outdo Biotech Co., Ltd. (Shanghai, China).

To address batch effects and ensure data consistency, we took several measures. First, we included internal control samples in each sequencing batch to monitor and calibrate the sequencing process. Second, we employed batch correction techniques during data preprocessing, using the Harmony algorithm within the CellRanger_V5.0.0 software suite to harmonize the data across different batches. This approach helped to mitigate technical variations and batch-specific biases, providing a more accurate representation of the biological signals. Data comparison, gene quantification, and cell identification were executed utilizing CellRanger_V5.0.0 software, applying stringent filtering thresholds for each sample. These criteria included UMI counts ≤ 27,009, gene number per cell ranging from 200 to 5,368, mitochondria content ≤ 20%, and ribosome distribution ≤ 57.5%. After filtering, the data was preprocessed and normalized to facilitate comparative analysis. Subsequent cluster analysis was performed on the normalized dataset using the Louvain algorithm, while the UMAP algorithm was employed for data visualization. Furthermore, Gene Set Variation Analysis (GSVA) was applied to explore the functional attributes of the cells.

### Cell lines, plasmid construction, and transfection

MDA-MB-231, 4T1, and HEK293T cells were obtained from FuHeng Cell Center (Shanghai, China). Human breast cancer MDA-MB-231 cells were cultured in complete L-15 medium. Complete RPMI 1640 was used to grow murine breast cancer 4T1 cells.

*ATG5* knockdown MDA-MB-231 and 4T1 cells were constructed as previously described 19. Briefly, ATG5 siRNA (human: 5-TCAGCTCTTCCTTGGAACATCACAGTACA-3, mouse: 5-GCUUCGAGAUGUGUGGUUUTT-3) was subcloned into the pLVX-ShRNA2-Puro plasmid, and plasmids were transfected into HEK293T cells with psPAX2 and pMD2G plasmids using Lipofectamine 3000 (Invitrogen) for virus packaging. Then MDA-MB-231 or 4T1 cells were infected with ATG5 knockdown lentvirus for 48 h (MOI: 10 for MDA-MB-231 and 4T1) and positive cells were selected with puromycin (1 μg/mL for MDA-MB-231, 3 μg/mL for 4T1). Transfection efficiency was determined by western blotting and immunocytochemistry as shown in Fig. S12.

### Flow cytometry

Cells were treated with equal volumes of drugs or dimethyl sulfoxide for 24 h. After diluted to 1×10^5^ cells/mL, the cells were blocked in 2% normal mouse serum (36118ES03, Yeasen) for 20 min, then incubated with antibodies for 30 min at 4°C in the dark. For tumor and spleen tissues, single-cell suspensions were prepared according to the previous literature 26. Briefly, tissues were dissected from mice and digested in an L-15 digestion medium containing 1 mg/mL collagenase IV (2091, BioFroxx), 50 U/mL DNase I (1121, BioFroxx), and 5 mM CaCl_2_ (C4901, Sigma-Aldrich) on a shaker at 24°C for 1 h. The solution was filtered through a 70 μm cell strainer (352350, Corning). Red Blood Cell Lysis Buffer (R1010, Solarbio) was used to lyse erythrocytes. The flow cytometry experiments were performed using a BD LSRFortessa and NovoCyte flow cytometer, and the data were analyzed using the FlowJo_V10 software.

### Western blotting

Cells were treated with the drugs for 24 h, followed by trypsin digestion and lysis for protein extraction. Proteins were isolated and then transferred onto a polyvinylidene difluoride membrane (GVWP02500, Millipore). The blocking buffers (P0252, Beyotime) were used to block the membranes for 30 min at 24°C. Next, the membranes were incubated with the primary antibody at 4°C overnight and the secondary antibody for 1 h at 24°C. The protein bands were visualized using a Gel Doc TMXR+ System (Bio-Rad) and normalized to the reference protein (β-actin).

### Immunocytochemistry

Cells (1×10^5^) were cultured on coverslips in 24-well plates and treated with drugs for 24 h. After washing thrice, the cells were fixed in 4% paraformaldehyde (BL539A, Biosharp) for 15 min. Permeabilizing was performed with Triton X-100 (1139, BioFroxx) for 20 minutes. Next, we blocked the cells with normal goat serum (ZLI-9022, ZSGB-BIO) for 1 h. Primary and fluorescently conjugated secondary antibodies were each incubated for 1 h at 24°C in the dark. Cell slides were sealed with an antifade mounting medium containing DAPI (P0131, Beyotime) and observed under an AX10 IMAGER A2 fluorescence microscope. The mean fluorescence was evaluated using ImageJ software.

### Immunohistochemistry (IHC) staining

Patient samples were obtained from the Breast Cancer Center and the Department of Pathology, Southwest Hospital, Army Medical University (Chongqing, China), with strict adherence to ethical guidelines and informed consent procedures. A total of 20 TNBC patients diagnosed between 2015 and 2019 were included in this study. All patients were treatment-naïve at the time of sample collection, with no prior exposure to chemotherapy, radiotherapy, immunotherapy, or targeted therapy before surgical resection. Clinicopathological characteristics, including age, tumor size, lymph node status, histological grade, and Ki67 index, were collected from medical records and are summarized in Table S1.

Immunohistochemistry (IHC) staining was performed using the SP kit (PV-9000, ZSGB-BIO), following the instructions. The IHC staining intensity (SI) was scored as 0, negative; 1, weakly positive; 2, positive; and 3, strongly positive, and the percentage of positive cells (PP) was counted as 0, <5%; 1,6-25%; 2, 26-50%; 3, 51-75%; and 4, >75%. The final IHC staining score was calculated by multiplying the SI with the PP. All IHC scoring was performed independently by two pathologists who were blinded to the experimental group allocation and patient clinical information. Discrepancies between the two scorers were resolved by consensus discussion with a third investigator. The mean fluorescence was measured using the ImageJ software. Based on the IHC staining scores, the patient samples were stratified into LC3 low-expression (scores ≤8) and high-expression (scores >8) groups.

### Statistical analysis

All data are expressed as the mean ± standard deviation. For comparison of two groups, Student's t-test was used if the variance is homogeneous, Mann-Whitney U test was used if the variance is not homogeneous. For pair-wise comparisons, one-way analysis of variance and the post-hoc Bonferroni test was used. For correlation analysis, Pearson correlation test was used. Values of *P* < 0.05 was considered significant. GraphPad Prism 8.0 was used for statistical analysis.

## Results

### PS-T potentiates the therapeutic efficacy of anti-PD-L1 antibodies in TNBC via activating immune cells

**Figure 1:**
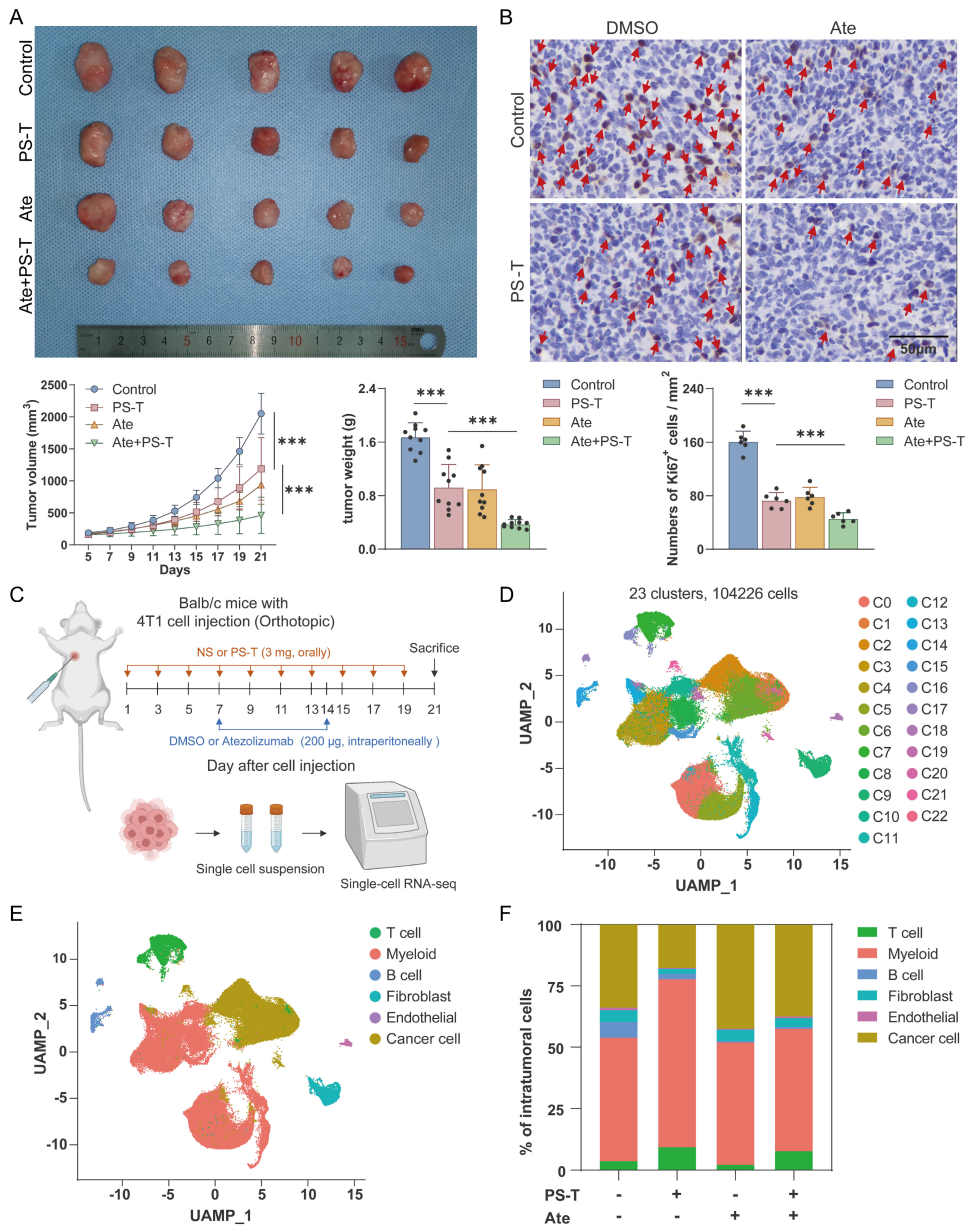
** PS-T enhances the treatment efficacy of anti-PD-L1 antibodies in TNBC. (A)** 4T1 tumor-bearing mice were photographed after treatment with isotonic sodium chloride solution (control), PS-T (3 mg, every two days), or atezolizumab (200 μg, once a week) alone or in combination with both (n=10). Tumor volume and weight were measured and analyzed statistically and are presented as a histogram (*bottom*). **(B)** Representative immunohistochemical staining for Ki67 in mouse breast tumor tissues under different conditions (n=6). Arrows indicate Ki67-positive cells (brown nuclear staining). Scale bars = 50 μm. **(C)** Schematic representation of the scRNA-seq experimental protocol. **(D)** UMAP clustering of 23 subgroups.** (E)** UMAP plot of 6 subsets after cell annotation. **(F)** The cell type distribution across four groups of tumor tissues. (mean ± standard deviation; **P < 0.01; ***P < 0.001).

In order to investigate the synergistic effect of anti-PD-L1 antibodies and PS-T, we constructed 4T1 mouse breast cancer models and treated the mice with indicated drugs. PS-T did not induce apparent drug-induced acute toxicity in Balb/c mice (Fig. S1). The combination of atezolizumab (Ate) and PS-T exhibited enhanced inhibition on tumor growth and cell proliferation, compared to that seen in the single administration group (Fig. 1A-B). We then performed single-cell RNA sequencing on the xenograft tumors (Fig.1C). Transcriptomes of 23193, 33703, 23430, and 23900 cells were obtained in the control, PS-T, Ate, and Ate+PS-T-treated tumors, respectively (Fig.1D). Six major cell types were identified, including cancer cells, T cells, myeloid cells, B cells, fibroblasts, and endothelial cells (Fig. 1E). The distribution of the cell types within the tumors is shown in Fig. 1F. GSVA enrichment of cancer cells suggested that PS-T stimulates IFN-α and IFN-γ response, and promotes complement, inflammatory response, TNF-α/NF-kB, and IL-2/STAT5 signaling, exhibiting an activation of antitumor immunity in TNBC (Fig. S2). Besides, we noticed a dynamic alteration in the Col13a1+ and Col14a1+ fibroblast populations across the treatment groups. PS-T treatment led to a prevalence of Col14a1+ fibroblasts, whereas Col13a1+ fibroblasts were more abundant in the control and Ate-treatment groups (Fig. S3A-D). Although the less pronounced proportional change than other cell types, endothelial-mediated enhancement of tumor immunity was observed following PS-T treatment (Fig. S3E-H).

**Figure 2:**
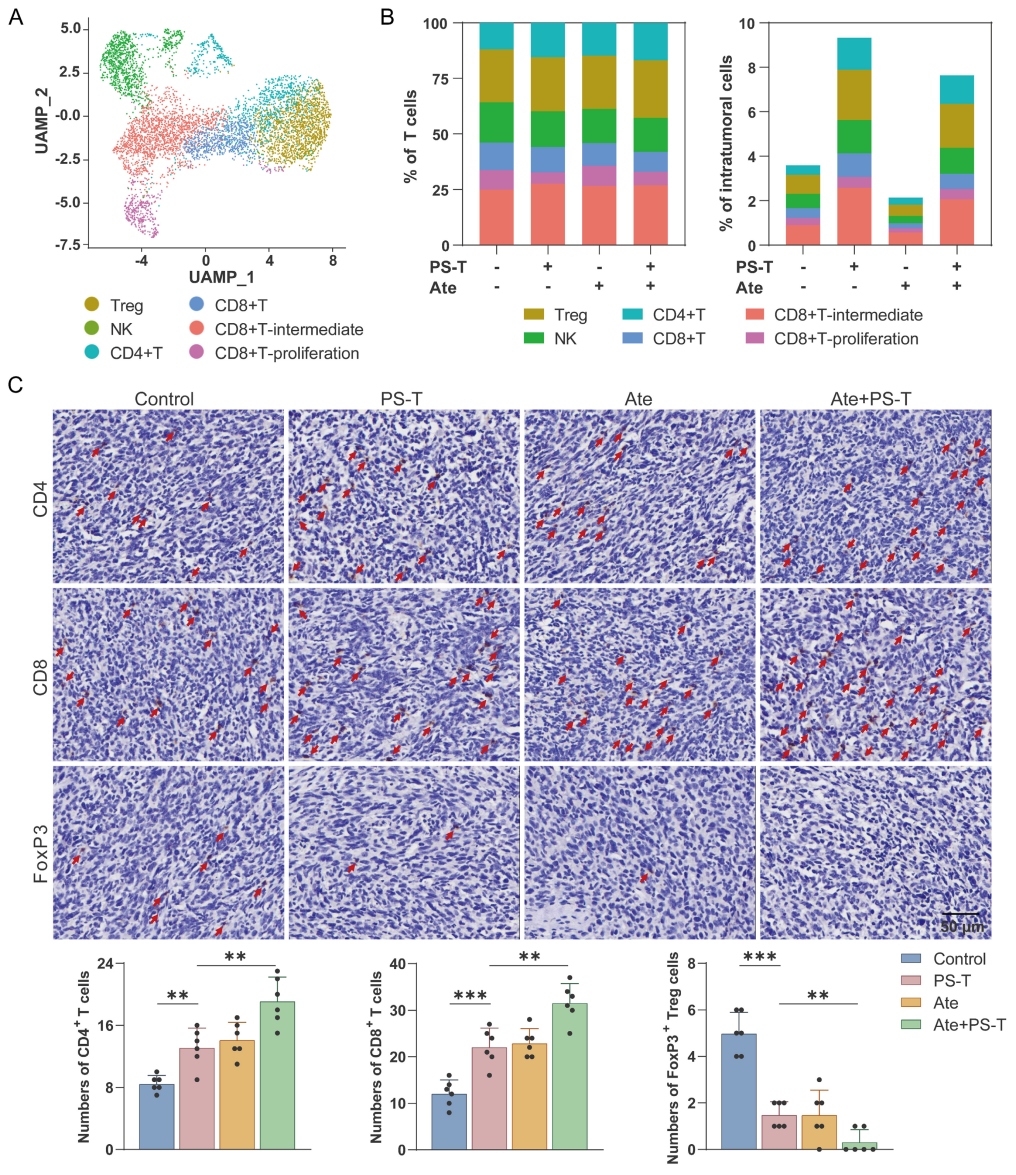
** PS-T promotes the infiltration and activation of T cells mediated by anti-PD-L1 treatment. (A)** UMAP clustering of tumor-infiltrating T cells identified 6 clusters (Treg, NK, CD4+T, CD8+T, CD8+T-intermediate, and CD8+T-proliferation).** (B)** The ratio (*left*) and number (*right*) of T cell subsets.** (C)** Representative images of CD4^+^ and CD8^+^ T cells and FoxP3^+^ Tregs in mice treated with PS-T, anti-PD-L1 antibody, and a combination of both. The histogram (*right*) depicts the number of positive cells in each group (n=6). Scale bars = 50 μm. The data were combined based on two independent experiments. (mean ± standard deviation; **P < 0.01; ***P < 0.001).

Due to the elevated tumor-infiltrating immune cells contributes to better prognosis for TNBC tumors 27, 28, we next investigated the role of immune cells in PS-T-mediated enhancement of antitumor effect of anti-PD-L1 antibodies, and observed significant changes in the proportion of immune cells after Ate and PS-T treatment (Fig. 1F). The percentage of intratumoral T, NK, CD4^+^T, CD8^+^T, CD8^+^T-intermediate, and CD8^+^T-proliferation cells increased after PS-T treatment (Fig. 2A-B and S4A-E). GSVA analysis showed activation of multiple immune-related pathways in T cells, including TNF-α/NF-kB, Notch, IFN-α, IFN-γ, and IL-2/STAT5 signaling, complement, and inflammatory response (Fig. S4F). Moreover, Ate and PS-T combination treatment increased the number of CD4^+^ and CD8^+^ T cells, while decreasing the number of FoxP3^+^ Tregs compared to the single-agent groups (Fig. 2C and S4G-H). Additionally, PS-T significantly increased the proportion and immunological activity of intratumoral myeloid cells, whether or not it was combined with anti-PD-L1 antibodies (Fig. S5A-C). The clustering and GSVA results revealed that PS-T treatment diminished the immunosuppressive myeloid-derived suppressor cells (MDSCs), and activated the immunoactivating dendritic cells (DCs) (Fig. S5D-G).

### PS-T reverses the immunosuppressive microenvironment in TNBC

**Figure 3:**
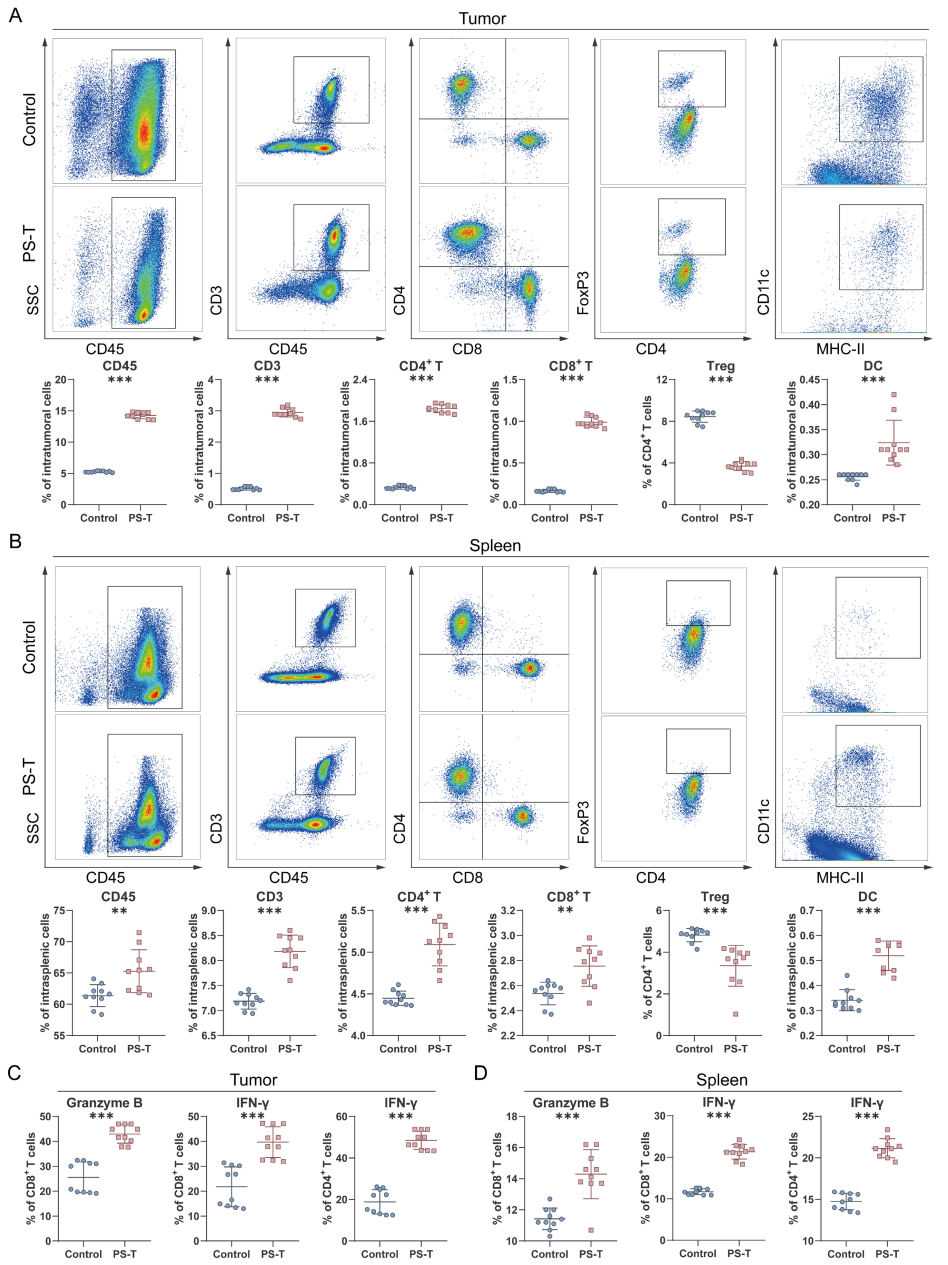
** PS-T affects the tumor immune microenvironment in TNBC. (A-B)** Representative plots (*up*) of CD45^+^, CD3^+^ T, CD4^+^ T, and CD8^+^ T cells as well as regulator T cells (Tregs) and dendritic cells (DCs) in the tumor (A) and spleen tissue (B) of 4T1 mice treated with or without PS-T. The proportions are shown in the statistic graph (*bottom*) (n=10). **(C-D)** The proportions of IFN-γ^+^ and granzyme B^+^ cells in tumor-infiltrating CD4^+^ T and CD8^+^ T cells in the breast tumor (E) and spleen (F) (n=10). The data were combined based on two independent experiments. (mean ± standard deviation; **P < 0.01; ***P < 0.001).

We next profiled the tumor infiltrating immune cells by flow cytometry. The gating strategy for flow cytometry analysis is illustrated in Fig. S6. In PS-T-treated group mice, a significantly higher number of intratumoral CD45^+^ cells was observed compared to the control group (Fig. 3A). Besides, antitumor T cell immune responses were detected, marked by an increase in CD3^+^ T cells, CD4^+^ T cells, CD8^+^ T cells, and MHC-II^+^ CD11c^+^ DCs, along with a decrease in CD4^+^ FoxP3^+^ Tregs (Fig. 3A). Immune cells within spleen tissue were then examined to assess whether PS-T treatment could impact peripheral immune organs. As depicted in Fig. 3B, the changes of immune cells in the spleen exhibited similar results to those in the tumor.

In addition to frequency of immune cells, we also investigated whether PS-T could enhance T cell function. The percentage of activated cytotoxic T cell subgroups (granzyme B^+^ CD8^+^ T, IFN-γ^+^ CD8^+^ T, and IFN-γ^+^ CD4^+^ T) was markedly elevated in breast tumors and spleens of PS-T-treated mice (Fig. 3C-D and S7). To further characterize the functional status of tumor-infiltrating T cells, we assessed the expression of exhaustion markers PD-1, LAG-3, and TIM-3 on CD8^+^ T cells. As shown in Fig. S8, PS-T treatment significantly reduced the frequency of PD-1^+^LAG-3^+^ and PD-1^+^TIM-3^+^ CD8^+^ T cells compared to the control group, indicating that PS-T alleviates T cell exhaustion. These findings suggest that PS-T not only enhances T cell activation but also mitigates T cell exhaustion, thereby contributing to the enhanced antitumor immune response in TNBC.

### PS-T promotes autophagic degradation of PD-L1 in TNBC

**Figure 4:**
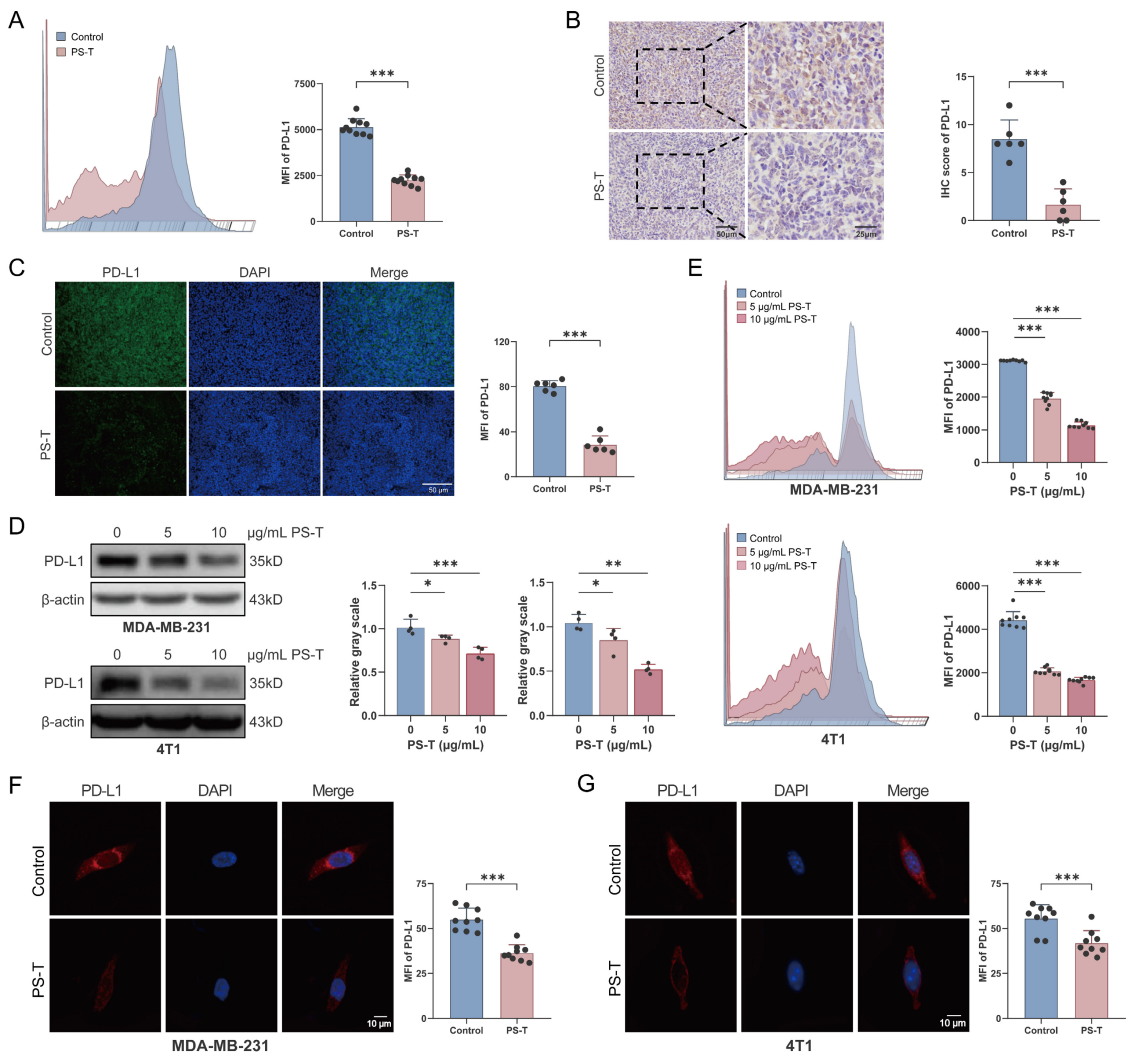
** PS-T downregulates PD-L1 expression in TNBC. (A-C)** The expression of PD-L1 in the breast cancer tissue of mice treated with or without PS-T was determined by flow cytometry (A) (n=9), immunohistochemical staining (B), and immunofluorescence staining (C) (n=6). Scale bars = 50 and 25 μm. **(D-E)** Western blotting (D) (n=3) and flow cytometry analysis (E) (n=9) of 0, 5, and 10 μg/mL treated 4T1 and MDA-MB-231 cells. **(F-G)** Representative fluorescent micrographs of PD-L1 stained 4T1 (F) and MDA-MB-231 cells (G) (n=9). The data were combined based on three (A, D-G) or two independent experiments (B-C). (mean ± standard deviation; *P < 0.05; **P < 0.01; ***P < 0.001).

Given that PS-T treatment promoted cancer cell-mediated immune response and reversed the immunosuppression of the tumor microenvironment, thereby enhancing the inhibitory effects of anti-PD-L1 antibodies on TNBC (Fig. S2), we investigated the changes in PD-L1 expression following *in vivo* treatment with PS-T. In the 4T1 mouse breast cancer xenografts, the mean fluorescence intensity (MFI) of PD-L1 diminished after PS-T administration (Fig. 4A). Histological examination of the tumor tissues revealed a significant suppression of PD-L1 protein expression post-PS-T treatment (Fig. 4B-C). Subsequently, we verified the downregulation of PD-L1 protein levels by PS-T* in vitro*. In both MDA-MB-231 and 4T1 cells, PD-L1 expression was inhibited by PS-T in a dose-dependent manner (Fig. 4D-G). To exclude the possibility that the observed reduction in PD-L1 expression was attributable to non-specific cytotoxicity, we assessed cell viability in MDA-MB-231 and 4T1 cells treated with PS-T at concentrations of 0, 5, and 10 μg/mL using CCK-8 assays. As shown in Fig. S9, PS-T did not induce significant cytotoxicity at these concentrations, confirming that the dose-dependent downregulation of PD-L1 reflects specific biological effects rather than general toxicity.

**Figure 5:**
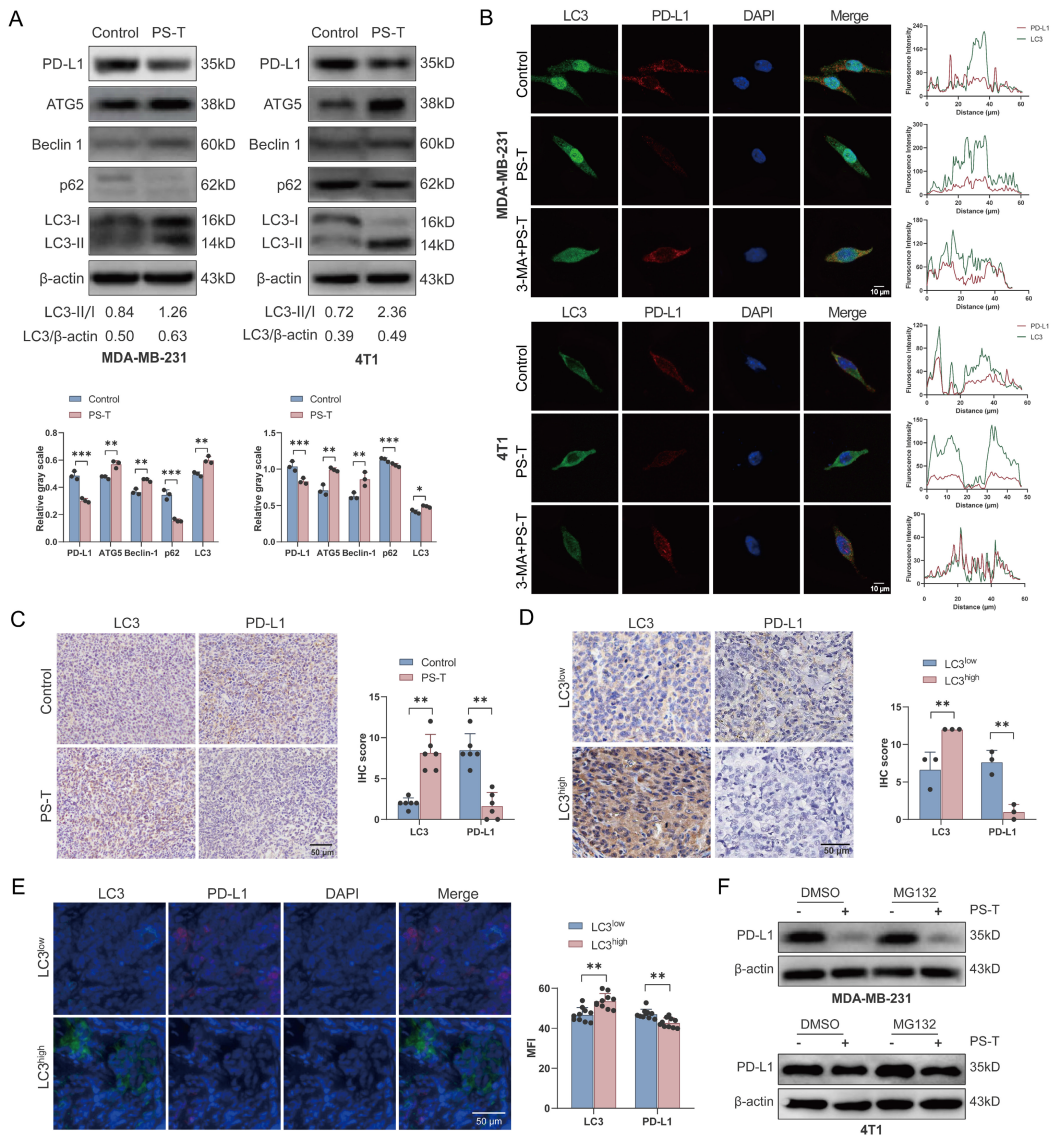
** PS-T regulates PD-L1 expression by inducing autophagy in TNBC. (A)** Expression levels of PD-L1 and autophagy-related markers in MDA-MB-231 (*left*) and 4T1 cells (*right*) treated with 5 μg/mL PS-T for 24 h (n=3). The grayscales were quantified using ImageJ software and calculated relative to β-actin levels. **(B)** The colocalization of LC3 and PD-L1 in MDA-MB-231 (*up*) and 4T1 cells (*down*) treated with or without PS-T was analyzed by immunofluorescence staining (n=9). Scale bars = 10 μm. **(C)** Representative images of LC3 (*left*) and PD-L1 (*right*) protein expression in the control and PS-T-treated mice (n=6). Scale bars = 50 μm. **(D-E)** The levels of LC3 (*left*) and PD-L1 (*right*) in TNBC patients were assessed by immunohistochemistry (n=3) (D) and immunofluorescence (n=10) (E). Scale bars = 50 μm. **(F)** The expression of PD-L1 in MDA-MB-231 (*up*) and 4T1 cells (*down*) treated with PS-T and/or MG132 (n=3). The data were combined based on three (A-B, D, F) or two independent experiments (C, E). (mean ± standard deviation; *P < 0.05; **P < 0.01; ***P < 0.001).

To explore the mechanisms by which PS-T downregulates PD-L1 expression, we performed RNA sequencing of TNBC cells treated with or without PS-T. However, there was no statistical differences in PD-L1 (*CD274*) transcript levels, suggests that PS-T modulates PD-L1 protein expression but not at the transcription level (Fig. S10). It is widely recognized that autophagy modulates PD-L1 expression 29, 30. We previously demonstrated that PS-T could significantly induce autophagy 19. In this study, we confirmed that PS-T reduced PD-L1 and p62 protein expression while enhancing the expression of key autophagy proteins, including ATG5, Beclin-1, and LC3 (Fig. 5A). To investigate the role of autophagy in PD-L1 degradation induced by PS-T, we observed the immunofluorescence staining of LC3 and PD-L1 using confocal microscopy. The results revealed that PD-L1 protein was degraded following PS-T treatment and co-localized with the LC3 protein (Fig. 5B). Immunohistochemistry analysis using 4T1 xenograft verified the upregulation of LC3 expression and downregulation of PD-L1 expression *in vivo* due to PS-T treatment, revealing a significant correlation between their expression (R^2^ = 0.6319, P = 0.0020) (Fig. 5C and S11A). Moreover, PD-L1 protein levels were found to be low in patients whose tumors exhibited high levels of LC3 expression (R^2^ = 0.8800, P = 0.0056) (Fig. 5D-E and S11B). In addition, we recognized that intracellular protein degradation is primarily mediated by two major pathways: the ubiquitin-proteasome pathway and the autophagy-lysosome pathway 31. Western blotting with proteasome inhibitor MG132 did not show increased PD-L1 level PS-T-treated cells, indicating that PS-T downregulates PD-L1 independently of the ubiquitin-proteasome pathway (Fig. 5F).

### Autophagy inhibition reverses the PS-T-induced activation of antitumor immunity in TNBC

**Figure 6:**
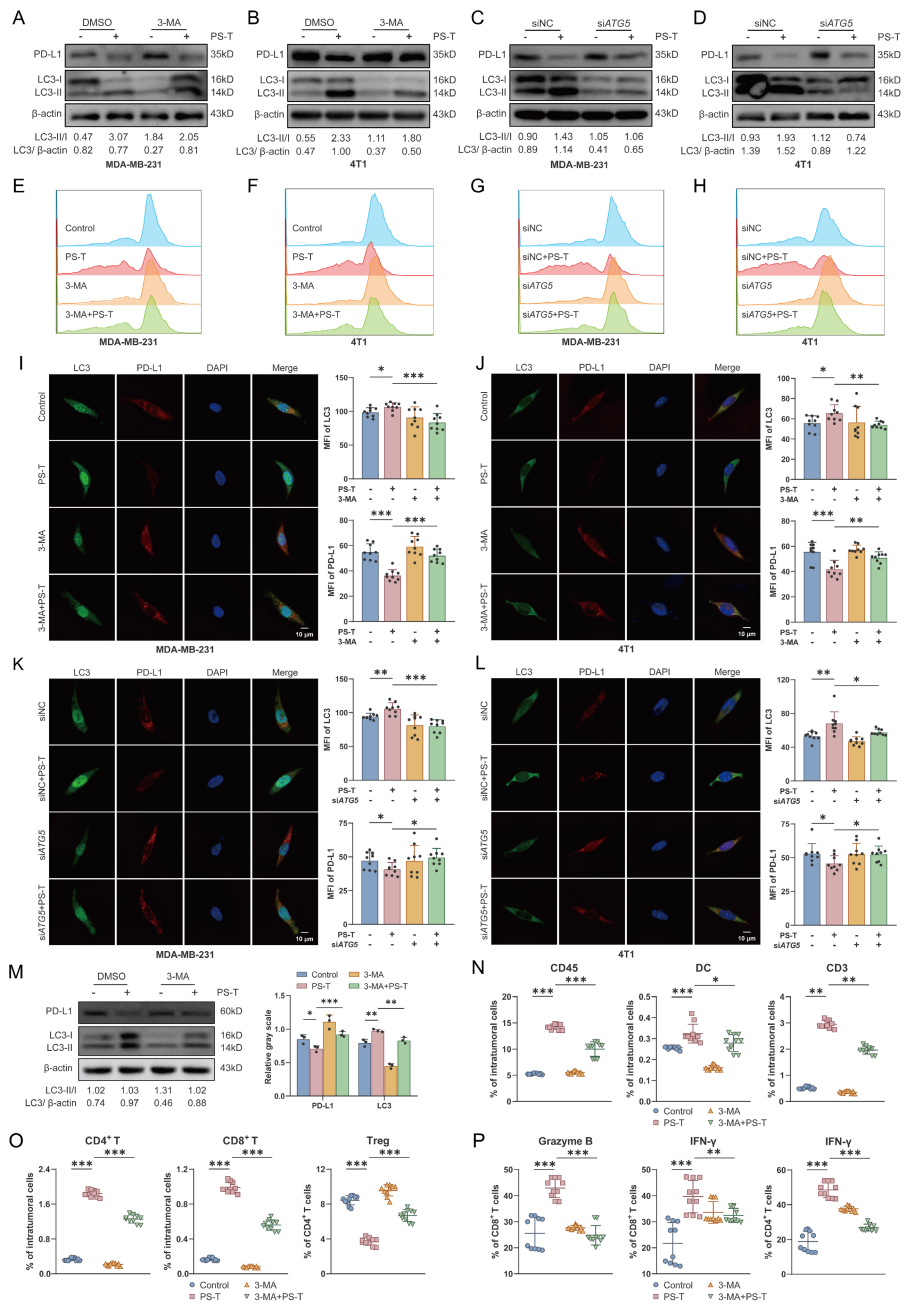
** Autophagy inhibition reverses the PS-T-induced activation of PD-L1-mediated T cell antitumor responses. (A-D)** Expression of PD-L1 and LC3 in MDA-MB-231 and 4T1 cells that autophagy was inhibited by 3-MA (5 mM) (A-B) and si*ATG5* (C-D) (n=3). **(E-H)** The levels of PD-L1 protein were examined by flow cytometry (n=9). **(I-L)** Confocal microscopy images of TNBC cells stained with PD-L1 (*red*) and LC3 (*green*). The MFI was quantified to indicate protein levels (*right*). Scale bars = 10 μm. **(M)** Mice were injected with 1×10^6^ 4T1 cells, randomly allocated into four groups, orally treated with 100 μL isotonic sodium chloride solution (control), 3 mg PS-T solution (PS-T) every two days, intraperitoneally administrated with 150 μL of 300 μg 3-MA solution (3-MA) every three days and co-treated with PS-T and 3-MA (3-MA+PS-T). The protein levels of PD-L1 and LC3 in the control, PS-T, 3-MA, and 3-MA+PS-T-treated tumors (n=3). **(N-O)** The concentration of CD45^+^, DC, CD3^+^ T, CD4^+^ T, and CD8^+^ T cells, Tregs in tumor tissues treated with PS-T (3 mg, every two days) and 3-MA (300 μg, every three days) alone or in combination (n=10). **(P)** Percentages of IFN-γ and granzyme B in tumor-infiltrating CD4^+^ T and CD8^+^ T cells in the untreated and PS-T-, 3-MA-, and 3-MA+PS-T-treated tumors (n=10). The data were combined based on three (A-M) or two independent experiments (N-P). (mean ± standard deviation; ns, not significant; *P < 0.05; **P < 0.01; ***P < 0.001).

We next used the upstream autophagy inhibitor 3-methyladenine (3-MA), lysosome inhibitor chloroquine (CQ), or knocked down the autophagy-related gene *ATG5* to inhibit autophagy in TNBC cells for subsequent experiments. The expression of ATG5 and LC3 was notably reduced in both si*ATG5*-4T1 and si*ATG5*-MDA-MB-231 cell lines (Fig. S12). Consistent with previous results, PS-T substantially lowered PD-L1 expression levels. However, the degradation was significantly reduced by inhibition of autophagy (Fig. 6A-L and S13).

To ascertain the role of autophagic PD-L1 degradation induced by PS-T in modulating tumor immune cell infiltration, we intraperitoneally administered 3-MA to 4T1 tumor-bearing mice to inhibit autophagy (Fig. 6M). Existing literature confirms that 3-MA does not impact breast tumor growth when used alone 32. Our data showed that 3-MA considerably reduced the PS-T-induced accumulation of CD45^+^, CD3^+^, CD4^+^ T, and CD8^+^ T cells and DCs, as well as the suppression of Treg infiltration (Fig. 6N-O). Moreover, PS-T treatment activated cytotoxic CD4^+^ and CD8^+^ T cells in breast tumors, with this activation being markedly restrained upon autophagy inhibition (Fig. 6P).

### Autophagic degradation of PD-L1 is associated with antitumor responses in TNBC patients

A total of 20 TNBC patients were enrolled in this study. All patients were treatment-naïve and underwent surgical resection without prior neoadjuvant therapy. The median age was 50.5 years (range, 28-78 years). Detailed clinicopathological characteristics, including tumor size, lymph node status, histological grade, and Ki67 index, are provided in Table S1.

**Figure 7:**
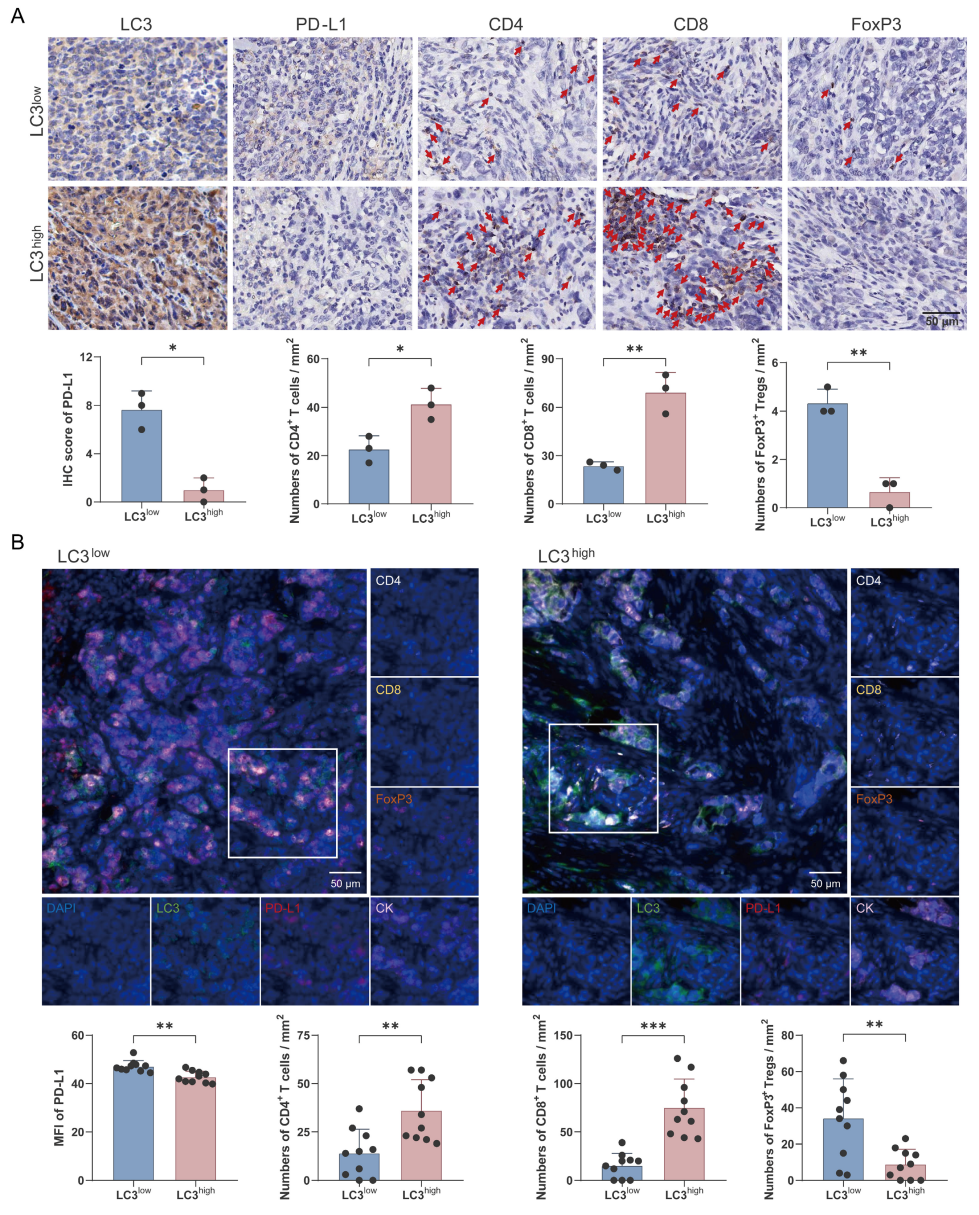
** Autophagic degradation of PD-L1 is associated with antitumor T cell responses in TNBC patients. (A)** Immunohistochemical staining of LC3, PD-L1, CD4, CD8, and FoxP3 in TNBC patients. IHC score of PD-L1 and numbers of CD4^+^ and CD8^+^ T cells and FoxP3^+^ Tregs were analyzed statistically and are presented as a histogram (*bottom*) (n=3). Scale bars = 50 μm. **(B)** Representative multicolor immunofluorescent staining images for LC3, PD-L1, CK, CD4, CD8, and FoxP3 in LC3^low^ (*left*) and LC3^high^ (*right*) TNBC patients (n=10). Scale bars = 50 μm. (mean ± standard deviation; *P < 0.05; **P < 0.01; ***P < 0.001).

To investigate the association between autophagy and antitumor immunity, the enrolled patients were divided into two groups based on LC3 protein expression levels. Our findings revealed that high LC3 expression was associated with notably lower PD-L1 levels (Fig. 7A). Futhermore, the high-LC3 group exhibited a significant increase in the infiltration of CD4^+^ and CD8^+^ T cells, whereas the number of FoxP3^+^ regulatory T cells was markedly decreased compared with the low-LC3 group (Fig. 7A). In additon, multicolor immunofluorescence staining was performed to further validate these observations, and the results confirmed that autophagic degradation of PD-L1 was positively correlated with enhanced antitumor immunity in TNBC (Fig. 7B).

## Discussion

Previous studies have demonstrated that polysaccharides derived from Traditional Chinese Medicine (TCM) possess robust immunomodulatory properties and exert antitumor effects 33-36. Among them, PS-T, the principal anticarcinogenic constituent of Huaier, has demonstrated efficacy in the treatment of various tumors, including gastric, liver, and breast cancer 16, 37-39. Despite existing research highlighting the stimulatory effect of PS-T on antitumor immunity, an understanding of its potential to enhance the sensitivity to immunotherapy and the underlying molecular mechanisms remains elusive. In this study, we performed single-cell sequencing alongside *in vitro* and* in vivo* experiments to demonstrate that PS-T could reverse the tumor immunosuppressive microenvironment and augment the efficacy of anti-PD-L1 antibodies in TNBC (Fig.1-2). The underlying mechanism involves PS-T enhancing the abundance and function of immune cells by autophagy-dependent degradation of PD-L1 in TNBC (Fig.3-7).

Lymphocytes are integral to the tumor microenvironment, with CD4^+^ T cells coordinating the immune response, CD8^+^ T cells (also known as cytotoxic T cells, CTL) executing the destruction of tumor cells through cytokines secretion and the release of cytotoxic particles containing perforin and granzymes 40, and Tregs contributing to tumor immune escape 41. In consonance with prior studies, our data revealed that PS-T promote CD4^+^ T and CD8^+^ T cells infiltration into the tumor and splenic microenvironment while inhibiting Tregs. Furthermore, we observed a significant increase in the proportion of cytotoxic CD4^+^ and CD8^+^ T cells, suggesting that PS-T activates T cells in TNBC (Fig. 2-3 and S4). In addition to promoting T cell activation, our data revealed that PS-T also alleviates T cell exhaustion, as evidenced by reduced expression of exhaustion markers including PD-1, TIM-3, and LAG-3 on tumor-infiltrating CD8^+^ T cells (Fig. S8). These findings highlight the potential of PS-T to reshape the T cell functional landscape in TNBC, promoting a more robust and sustained antitumor response.

Interestingly, the impact of PS-T on CD8^+^ T cells is still a matter of debate. Some argue that CD8^+^ T cell counts do not significantly rise following PS-T treatment 21, 42, possibly due to variations in gate strategies and final statistical indicators (Fig. S14). In our view, the cell proportion in tissues is a more reliable indicator of the immune system's particular status, rather than the percentages in T lymphocytes. Our data corroborate this perspective. Regarding Myeloid cells, which are the most abundant cell type in breast tumors, accounting for more than half of the tumors (Fig. 1F), we found that PS-T significantly decreased the counts of MDSCs in TNBC tumor-bearing mice and fostered the infiltration and activation of DCs (Fig. 3A-B and S5D-G). These findings suggest that PS-T could potentiate antitumor immune response in TNBC.

Additionally, we further explored the effects of PS-T on non-immune stromal cells. In agreement with the findings of Chen *et al*., PS-T reduced the number of intratumoral fibroblasts and those transitioning into myofibroblasts 43. We observed distinct shifts in fibroblast populations after PS-T treatment: Col13a1+ fibroblasts decreased significantly, while Col14a1+ fibroblasts increased, reversing the prevalence pattern seen in controls (Fig. S3A-D). The functional implications of these fibroblast subset shifts warrant further consideration. Col13a1, a member of the fibrillar collagen family, has been implicated in extracellular matrix remodeling and tumor progression. Conversely, Col14a1, which encodes a fibril-associated collagen with interrupted triple helices, has been associated with more organized extracellular matrix architecture and may facilitate immune cell infiltration. The observed PS-T-induced shift from Col13a1+ to Col14a1+ fibroblasts therefore may represent a functional transition toward a more immune-permissive stromal microenvironment. This phenotypic switch could contribute to the enhanced antitumor immune responses observed in our study by reducing physical barriers to immune cell infiltration and modulating stromal-derived immunosuppressive signals. Notably, the proportion of Col14a1+ fibroblasts increased specifically in the PS-T-treated groups, suggesting that this subset may play a supportive role in T cell recruitment or activation. While our current data do not establish a direct causal relationship between fibroblast subset shifts and immune remodeling, these findings provide a foundation for future mechanistic studies exploring how PS-T reprograms the stromal compartment.

PD-L1 is a vital immune checkpoint protein and a pivotal immunomodulator 44. PD-L1 expressed on tumor cells can inhibit T cell cytotoxicity and contribute to tumor immune evasion by binding to PD-1, thus leading to limited sensitivity to immunotherapies in tumors 45, 46. Our results indicate that PD-L1 protein expressing on tumor cells was downregulated following PS-T treatment (Fig. 4). To uncover how PS-T downregulate PD-L1 protein, we reviewed the literatures and noted that protein degradation mainly occurs through two systems: the proteasome and lysosomal-autophagy system 47. By using proteasome inhibitor MG132, we determined that PS-T does not degrade PD-L1 through the proteasome system (Fig. 5F). Besides, it has been reported that PS-T enhances autolysosome formation and autophagosome-lysosome fusion 48, and our data confirmed that PS-T promotes autophagy-dependent degradation of PD-L1 (Fig. 5A-E). PS-T has been shown to regulate the expression of LC3, ATG5, and Beclin-1, as well as the p62/SQSTM1 and MAPK/NF-κB pathways 19, 49, 50. Consequently, we posit that PS-T affects autophagy through multiple pathways, warranting further investigations to delineate the specific mechanisms by which PS-T promotes autophagy.

Interestingly, although PS-T significantly altered the abundance of myeloid cells, it did not significantly affect PD-L1 expression on these cell populations (Supplementary Table S2). This observation suggests that the immunomodulatory effects of PS-T on myeloid cells are likely mediated through mechanisms independent of PD-L1 regulation, such as promoting DC maturation and reducing MDSC accumulation as demonstrated in our scRNA-seq and flow cytometry data. The cell-type-specific effect of PS-T on PD-L1 expression, downregulating PD-L1 primarily in cancer cells rather than in immune cells, may be advantageous for combination immunotherapy, as it selectively reduces tumor cell-mediated immune evasion while preserving the physiological PD-L1 expression on antigen-presenting cells required for maintaining immune homeostasis. Future studies employing conditional knockout models or lineage-specific PD-L1 deletion could further elucidate the distinct contributions of cancer cell-intrinsic versus myeloid cell-intrinsic PD-L1 to the therapeutic efficacy of PS-T in combination with immune checkpoint blockade.

In this study, we report a novel finding that PS-T induces autophagic degradation of PD-L1 in TNBC cells (Fig. 5). The effect of PS-T on PD-L1 level was reversed when autophagy was inhibited using pharmacological inhibitors or small interfering RNA (Fig. 6). Our findings suggest that PS-T promotes the autophagic degradation of PD-L1, leading to the accumulation and activation of DCs, CD4^+^, and CD8^+^ T cells, and reducing the infiltration of Tregs (Fig. 3-5). Moreover, *in vivo* experiments demonstrated that the addition of an autophagy inhibitor mitigated the PS-T-mediated regulation of T cell immunity (Fig. 6M-P), which was further validated in TNBC patients (Fig. 7).

## Conclusion

PS-T enhances the antitumor immune response against TNBC by inducing autophagy-mediated degradation of PD-L1, thereby boosting the effectiveness of anti-PD-L1 antibody treatment. Our study establishes a compelling basis for future research, which should include validating these findings in patient-derived xenografts and organoid models to bridge the gap between preclinical observations and clinical applications. Such validations are crucial for assessing the therapeutic potential of PS-T and could inform the design of clinical trials for this novel intervention in TNBC treatment.

## Supplementary Material

Supplementary materials and methods, figures and tables.

## Data Availability

Additional Figures and associated Figure legends are provided in the supplementary text and are available online with the paper. The raw data of RNA sequencing has been uploaded to the GEO database [Accession No. GSE253683].
