# Supplementary material for: Huaier Polysaccharides Sensitize Anti-PD-L1 Therapy via Promoting Antitumor Immune Response in Triple-Negative Breast Cancer
Source: Int J Biol Sci. 2026 May 18;22(10):5509–24. doi: 10.7150/ijbs.133068 (PMC13215462; doi:10.7150/ijbs.133068)
Supplement: Supplementary file 1 — Supplementary materials and methods, figures and tables. [file ijbsv22p5509s1.pdf]

Supplementary Materials and Methods

Acute toxicity experiment

Six female Balb/c mice were randomly divided into control (3 mg PS-T, every other day) and experimental groups (30 mg PS-T, every other day), with three mice in each group. PS-T was dissolved in 100 μL of isotonic sodium chloride solution and administered via oral gavage. Body weight and food intake were measured daily for two weeks, and weekly changes were recorded. Mortality (if any) and abnormalities in the mice were also recorded. After the mice were sacrificed, organ weights and organ coefficients were measured, and the heart, liver, spleen, lung, and kidney tissues were prepared for H&E staining.

Cell viability assay

Cell viability was assessed using the Cell Counting Kit-8 (CCK-8, CK04, Dojindo, Japan). MDA-MB-231 and 4T1 cells were seeded in 96-well plates at a density of 5×10^3 cells per well and incubated overnight to allow attachment. Cells were then treated with PS-T at concentrations of 0, 5, and 10 μg/mL for 24 h. Following treatment, 10 μL of CCK-8 solution was added to each well and incubated for 2 h at 37 °C. The absorbance was measured at 450 nm using a microplate reader (Thermo Scientific). Cell viability was calculated as the percentage relative to untreated control cells. Each experiment was performed in triplicate and repeated at least three times.

Sequencing

We plated MDA-MB-231, HCC1806 and HCC1937 cells in 6-well plates and treated them with or without 50 μg/mL PS-T for 24 h. After digestion, the cells were stored in TRIzol reagent (5×10^6 cells per ml of TRIzol), transferred to DNase-free cryopreservation tubes, and stored at −80 °C. Sequencing was performed by MajorBio (Shanghai, China).

Supplementary Figures

A

Body weight gain, food and water consumption, and organ coefficient results of mice treated with PS-T

	Acute toxicity	
Parameters	Control	30 mg
Initial weight (g)	15.867±0.507	15.820±0.286^{ns}
One week (g)	17.067±0.621	16.950±0.439^{ns}
Two weeks (g)	18.103±0.681	18.070±0.479^{ns}
Body weight gain (g)	2.237±0.616	2.250±0.193^{ns}
Food intake (g/day)	3.213±0.291	3.153±0.153^{ns}
Heart coefficient	0.028±0.003	0.027±0.004^{ns}
Liver coefficient	0.262±0.006	0.255±0.006^{ns}
Spleen coefficient	0.018±0.001	0.018±0.001^{ns}
Lung coefficient	0.034±0.001	0.036±0.002^{ns}
Kidney coefficient	0.042±0.001	0.045±0.004^{ns}

Results are expressed as mean±SD (n=6)

^{ns} Not significant

B

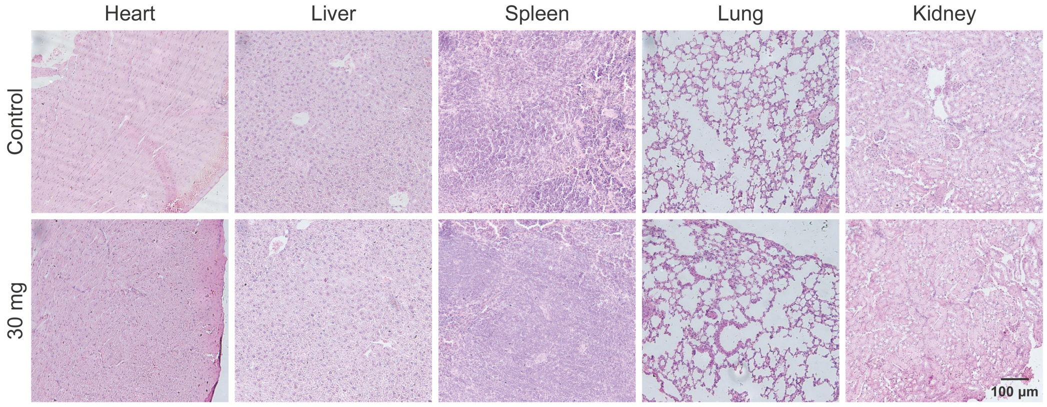

Fig. S1. Acute toxicity test of PS-T in mice

(A) Body weight gain, food and water consumption, and organ coefficient results of mice treated with 3 mg PS-T (control) and 30 mg PS-T (n=6). **(B)** Representative images of HE staining of heart, liver, spleen, lung, and kidney tissues from the control (*up*) and 30 mg PS-T group (*bottom*) (n=6). Scale bars = 100 μm. The data were combined based on two independent experiments. (mean ± standard deviation; ns, not significant).

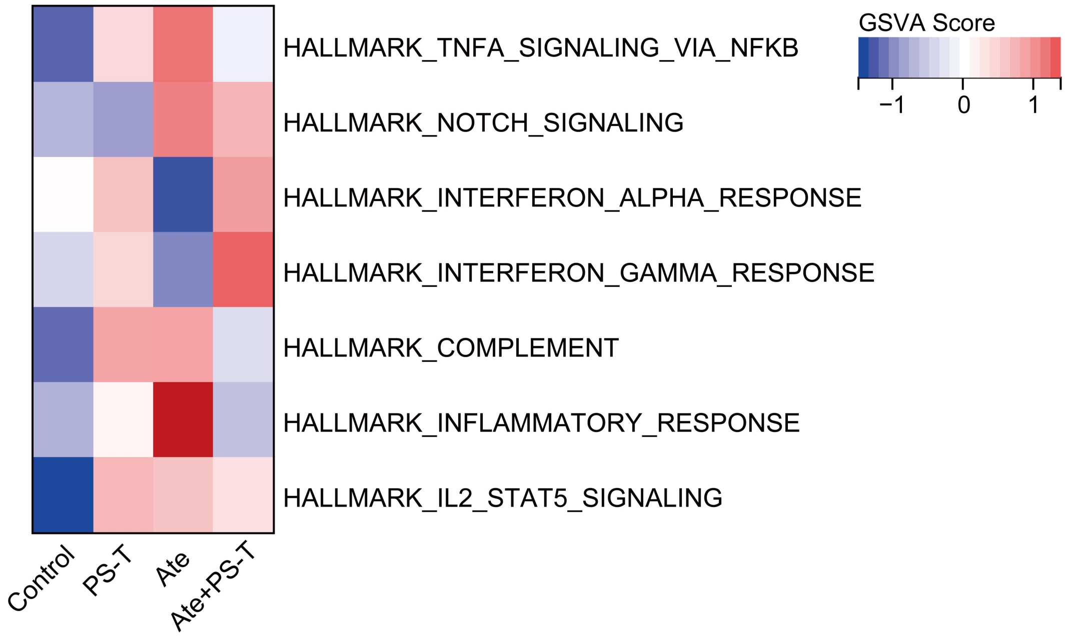

Fig. S2. GSVA analysis of cancer cells

GSVA analysis of cancer cells with TNF-α/NF-kB, Notch, and IL-2/STAT5 signaling, IFN-α, IFN-β, and inflammatory response, and complement.

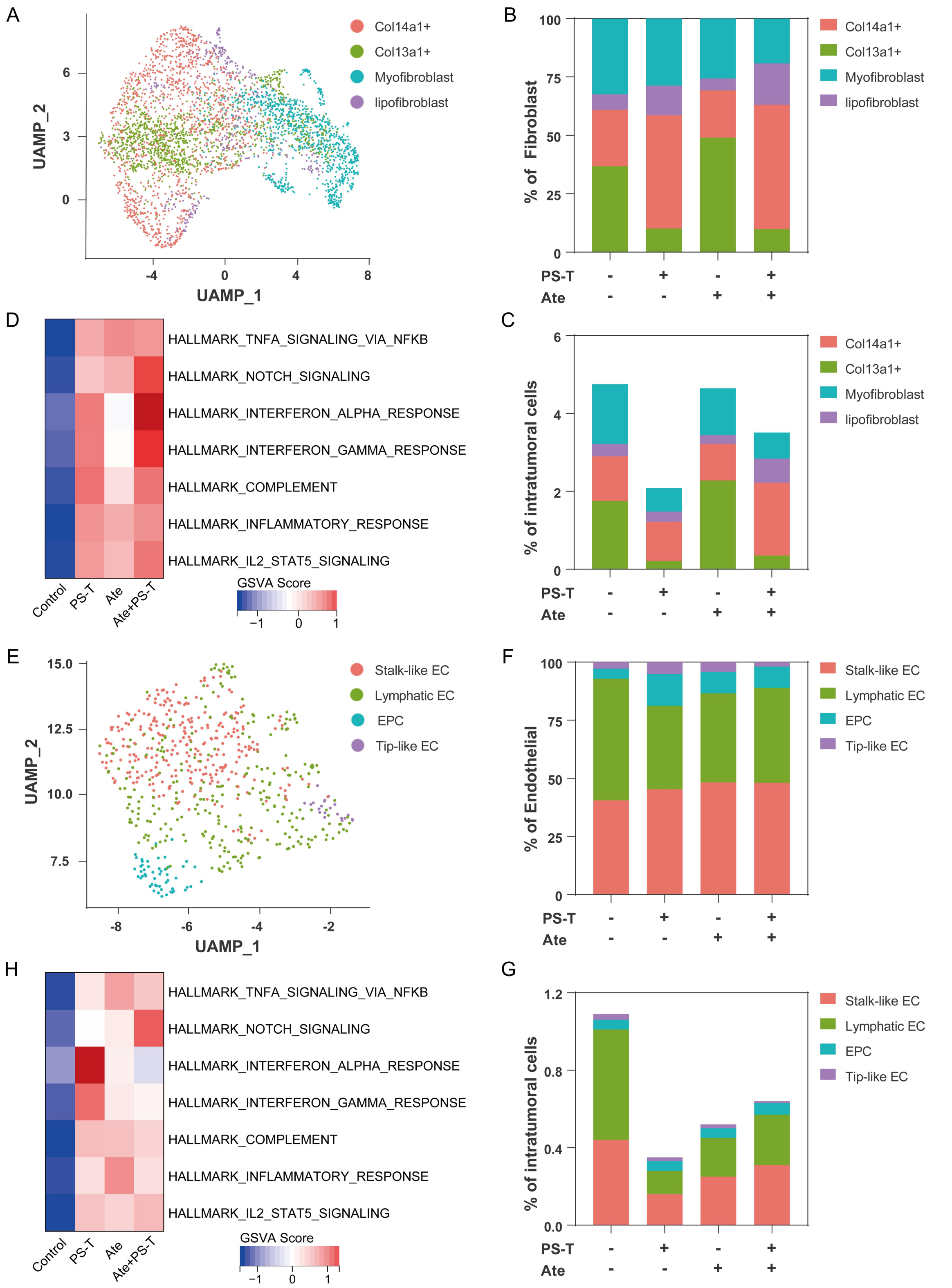

Fig. S3. Subclustering and GSVA analysis of fibroblasts and endothelial

(A) UMAP visualization of fibroblast subsets (Col14a1+, Col13a1+, Myofibroblast, and lipfibroblast).

(B-C) The proportions of subsets in fibroblasts (B) and intratumoral cells (C). **(D)** GSVA analysis of fibroblasts with immune-related pathways. **(E)** UMAP visualization of endothelial cell subsets (Stalk-like EC, Lymphatic EC, EPC, and Tip-like EC). **(F-G)** The proportions of endothelial cell subsets in endothelial (F) and intratumoral cells (G). **(H)** GSVA analysis of endothelial cells with immune-related pathways.

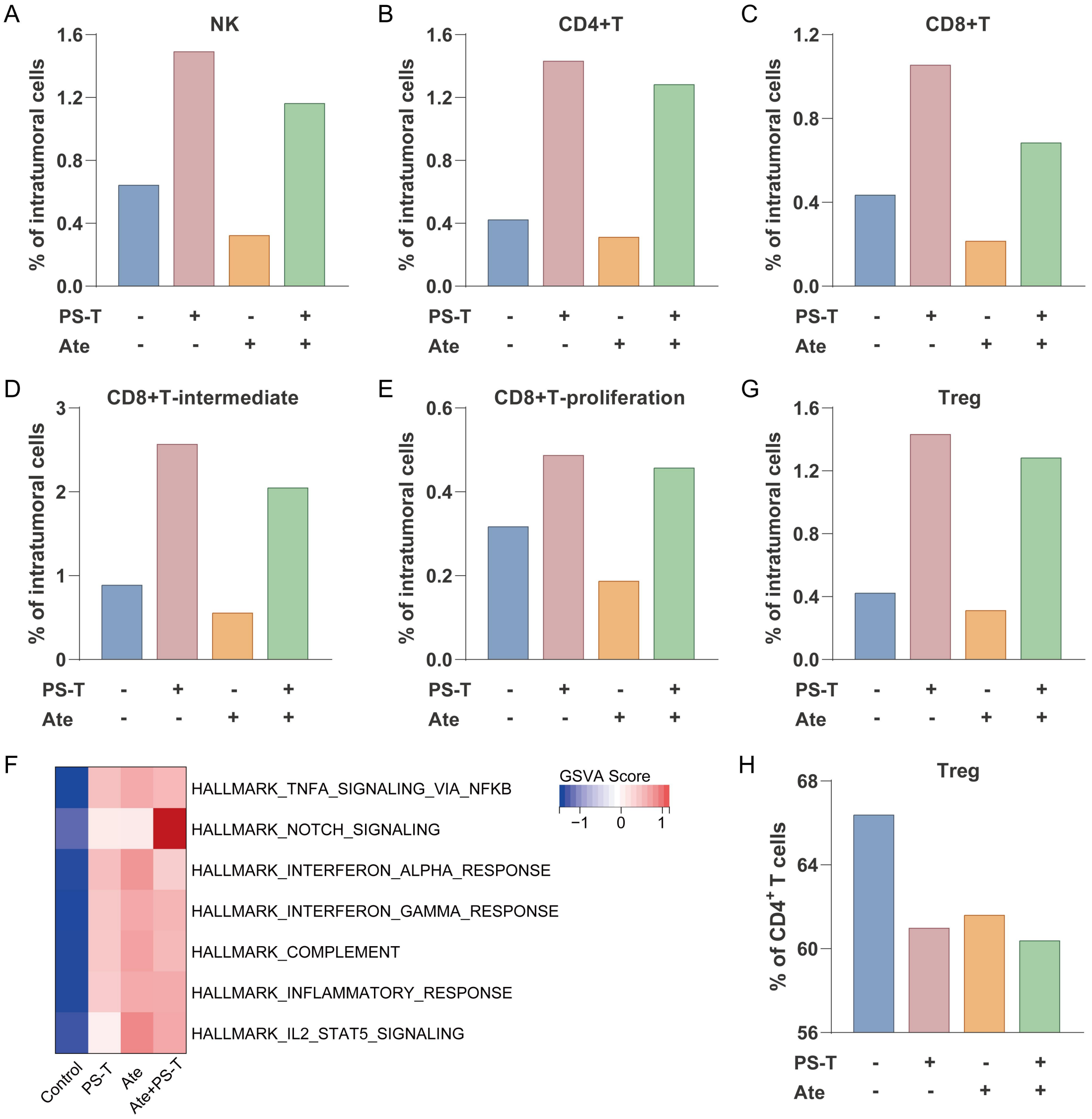

Fig. S4. Subclustering analysis of T cells

(A-E) The proportion of NK (A), $CD4^+$ T (B), $CD8^+$ T cells (C), $CD8^+$ T-intermediate (D), and $CD8^+$ T-proliferation cells (E) in intratumoral cells. **(F)** GSVA analysis of T cells with immune-related pathways. **(G-H)** The percentage of Tregs in intratumoral cells (G) and tumor-infiltrating $CD4^+$ T cells (H).

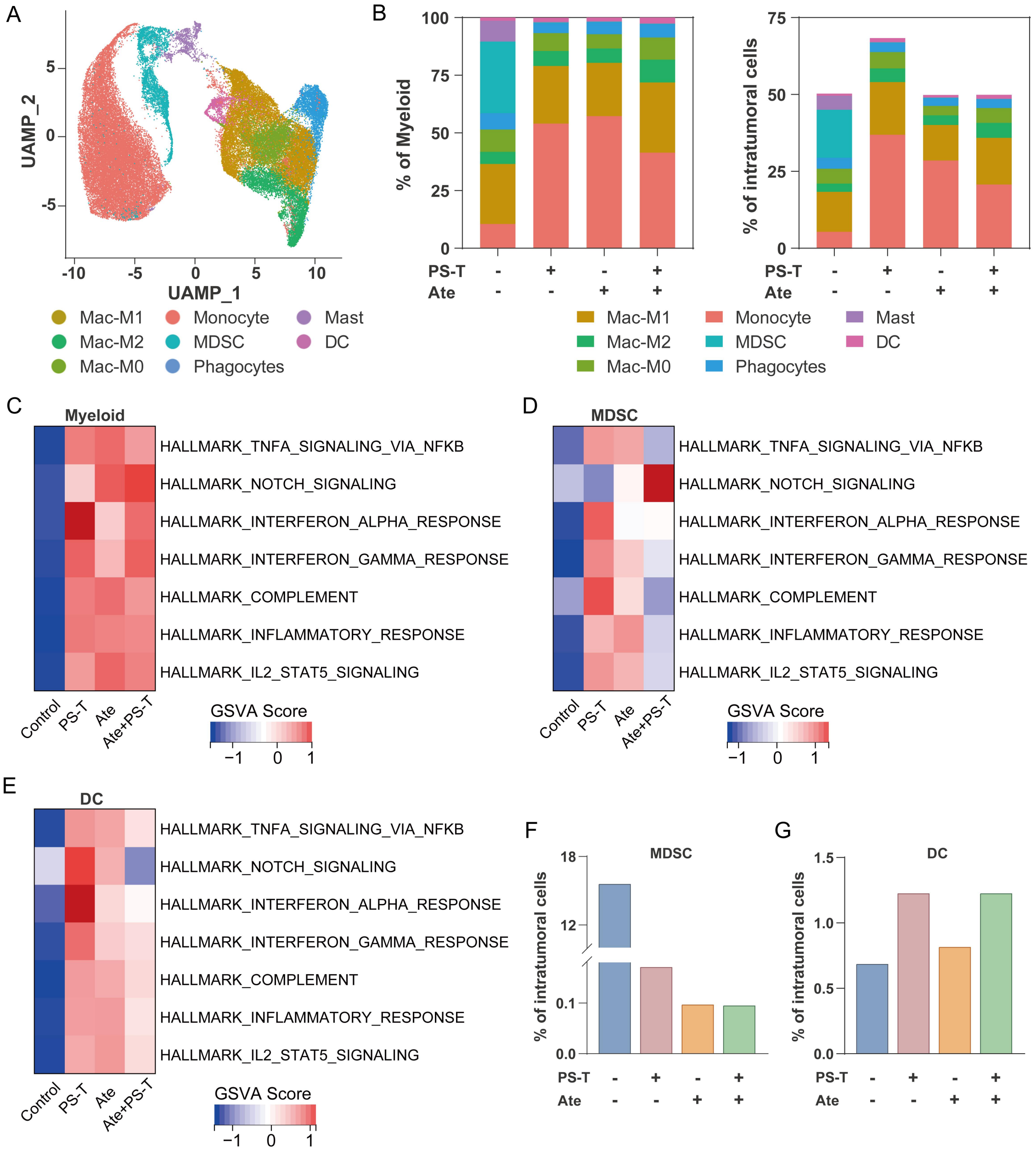

Fig. S5. Subclustering analysis of myeloid cells

(A) UMAP visualization of myeloid cell subsets (Mac-M1, Mac-M2, Mac-M0, Monocyte, MDSC, Phagocytes, Mast, and DC). **(B)** The proportions of myeloid cell subsets in myeloid (*left*) and intratumoral cells (*right*). **(C-E)** GSVA analysis of myeloid cells (C), MDSC (D), and DCs (E) with immune-related pathways. **(F-G)** The proportion of MDSC (F) and DCs (G) in intratumoral cells.

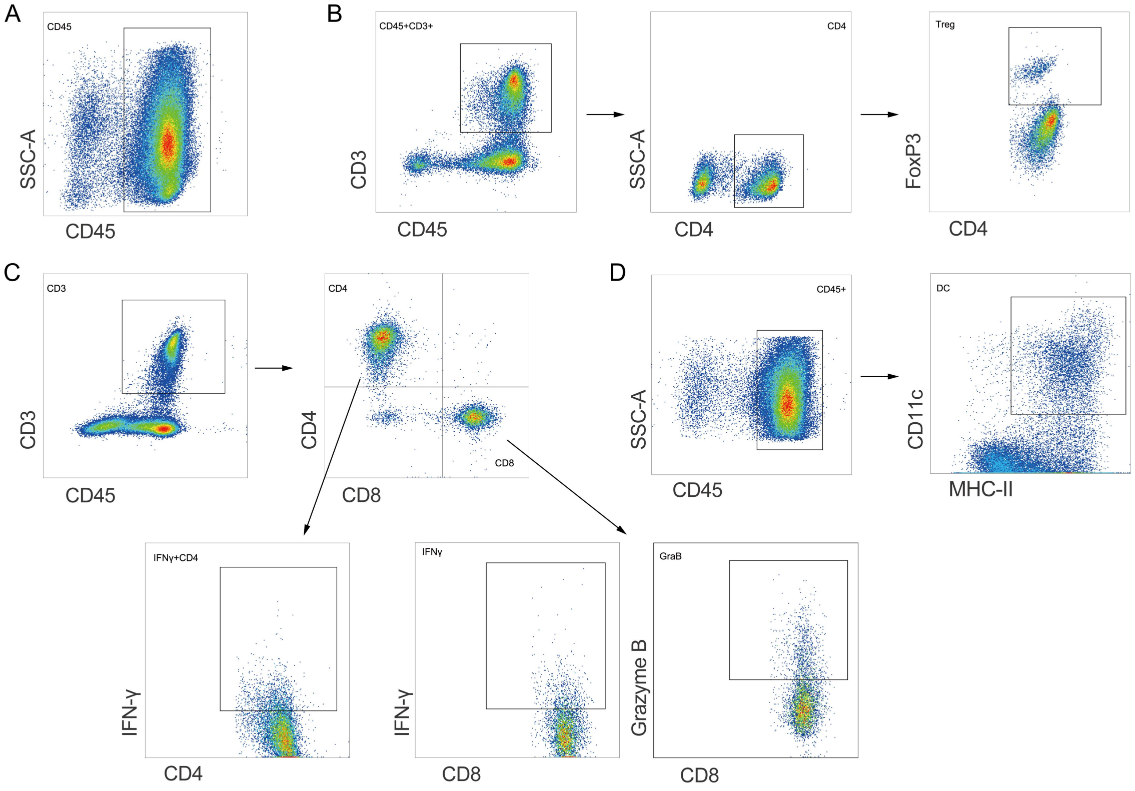

Fig. S6. Gating strategies of flow cytometry analysis

(A-D) Gating strategies of $CD45^+$ (A), regulatory T cells (Tregs) (B), $CD3^+$, $CD4^+$, and $CD8^+$ T cells, cytotoxic T cell subsets (granzyme B^+ $CD8^+$ T, IFN-γ^+ $CD8^+$ T, and IFN-γ^+ $CD4^+$ T) (C), and dendritic cells (DCs) (D).

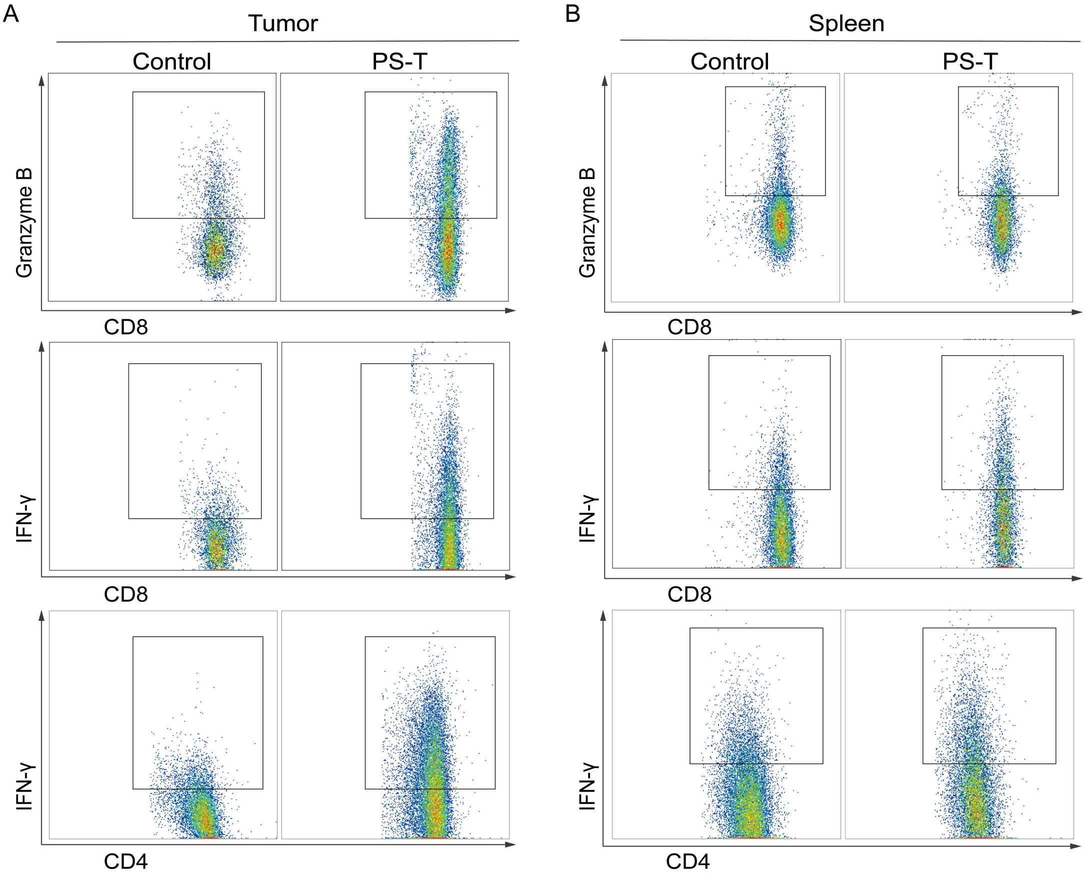

Fig. S7. PS-T enhances T cell function in TNBC

(A-B) Representative plots of IFN-γ and granzyme B in tumor-infiltrating $CD4^+$ T and $CD8^+$ T cells in the breast tumor (A) and spleen (B) (n=10).

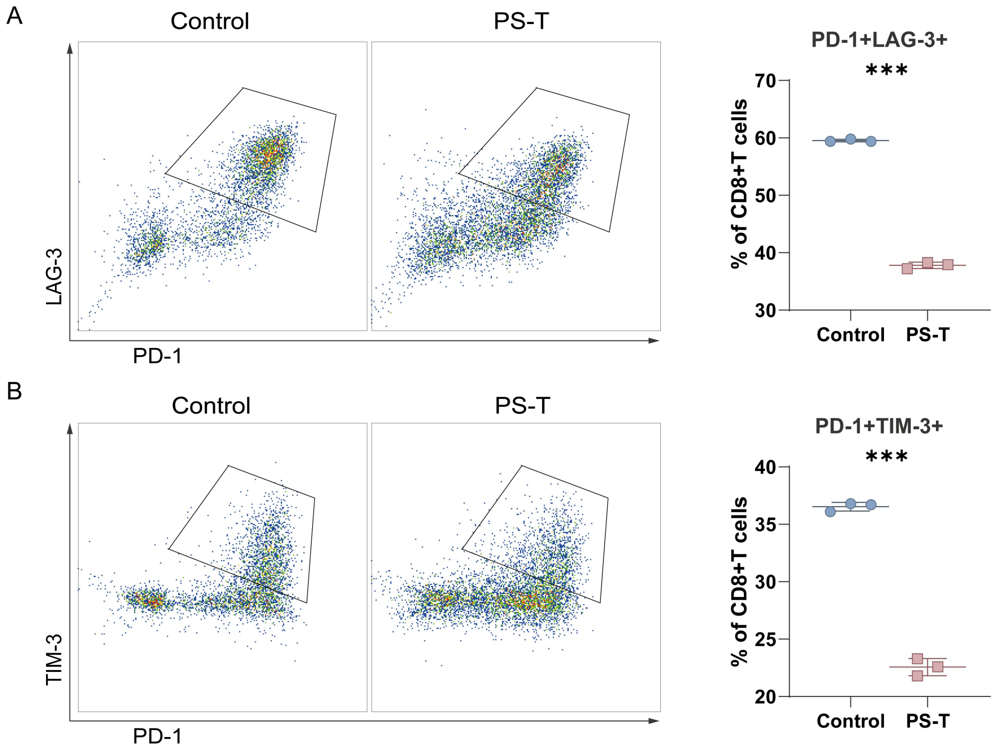

Fig. S8. PS-T alleviates T cell exhaustion in tumor-infiltrating CD8+ T cells

(A-B) Representative flow cytometry plots showing PD-1^+ LAG-3^+ (A) and PD-1^+ TIM-3^+ $CD8^+$ T cells (B) from 4T1 tumor-bearing mice treated with PS-T (n = 3). (mean ± standard deviation; ***P < 0.001).

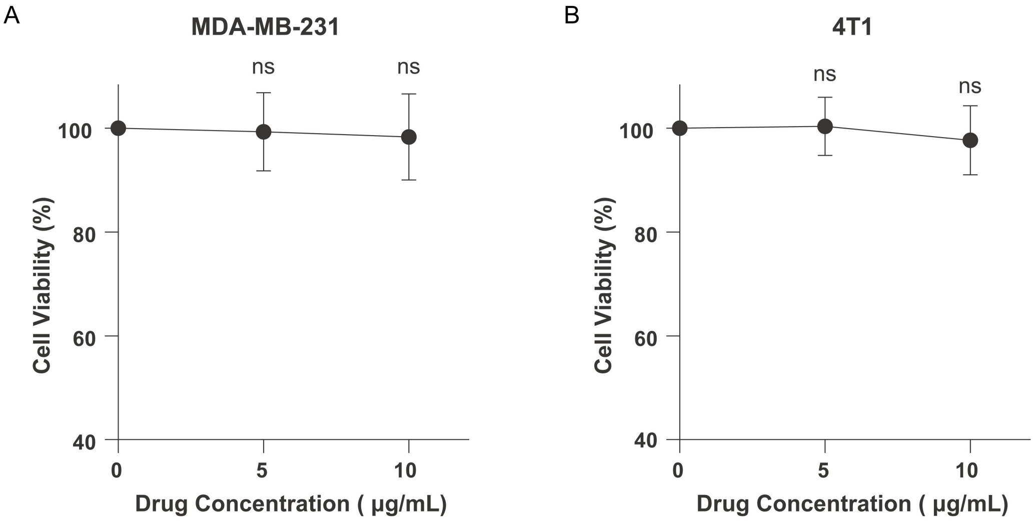

Fig. S9. Effect of PS-T on cell viability in TNBC cell lines

(A-B) MDA-MB-231 (A) and 4T1 (B) cells were treated with PS-T at indicated concentrations (0, 5, and 10 μg/mL) for 24 h, and cell viability was assessed by CCK-8 assay (n = 9). (mean ± standard deviation; ns, not significant).

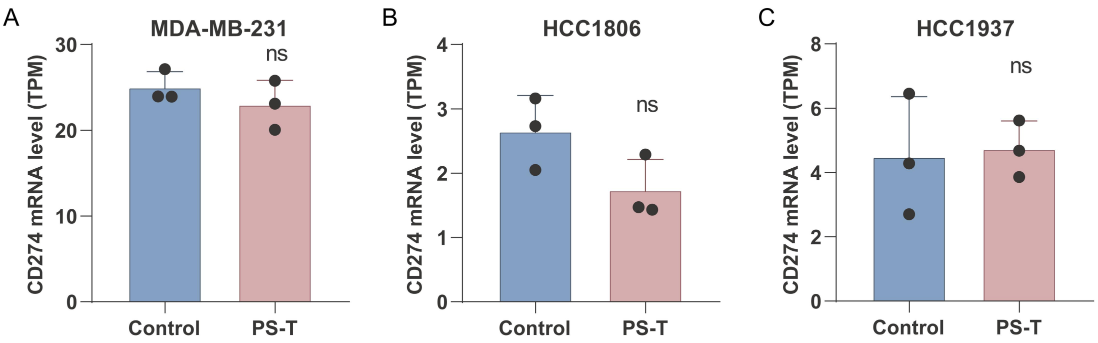

Fig. S10. PS-T modulates PD-L1 but not at the transcriptional level

(A-C) Transcriptome sequencing was performed to measure the TPM of *CD274* mRNA levels in the control and PS-T–treated MDA-MB-231 (A), HCC1806 (B), and HCC1937 cells (C). (mean ± standard deviation; ns, not significant).

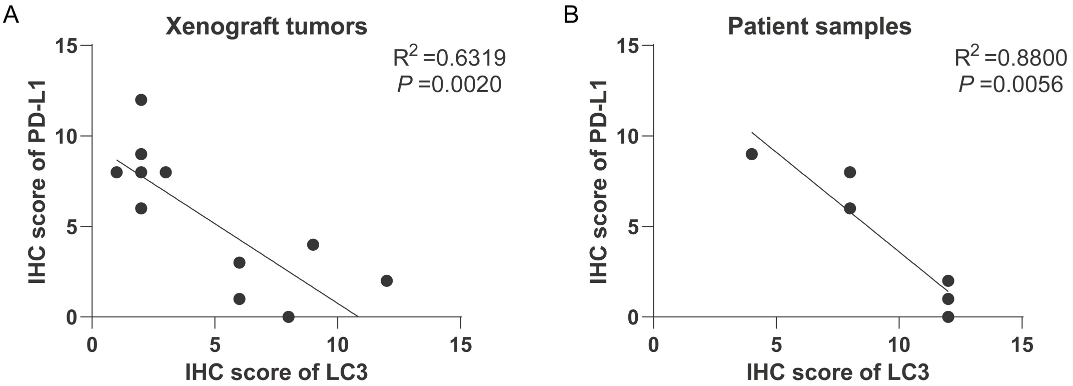

Fig. S11. Correlation analysis between LC3 and PD-L1 expression

(A) Correlation analysis of LC3 and PD-L1 expression in 4T1 tumor-bearing mice (R^2 = 0.6319, P = 0.0020). **(B)** Correlation analysis of the IHC score of LC3 and PD-L1 in TNBC patients (R^2 = 0.8800, P = 0.0056). The Pearson correlation test was used for the statistical analysis. The data were combined based on two independent experiments.

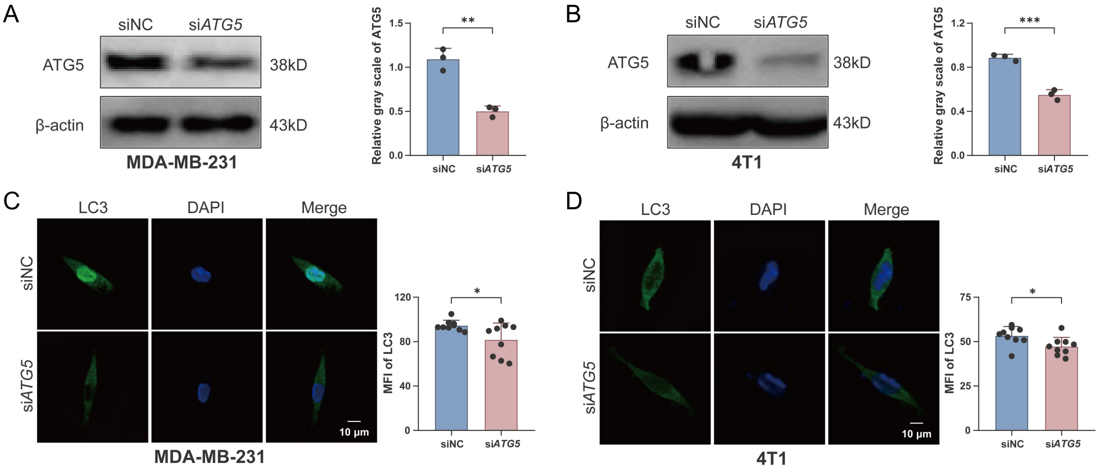

Fig. S12. Verification of ATG5 knockdown effect in TNBC cell lines

(A-B) The expression levels of ATG5 in MDA-MB-231 (A) and 4T1 cells (B) were analyzed using western blotting (n=3). **(C-D)** The expression levels of LC3 in MDA-MB-231 (C) and 4T1 cells (D) were evaluated by immunocytochemistry (n=9). The data were combined based on three independent experiments. (mean ± standard deviation; ***P* < 0.01; ****P* < 0.001).

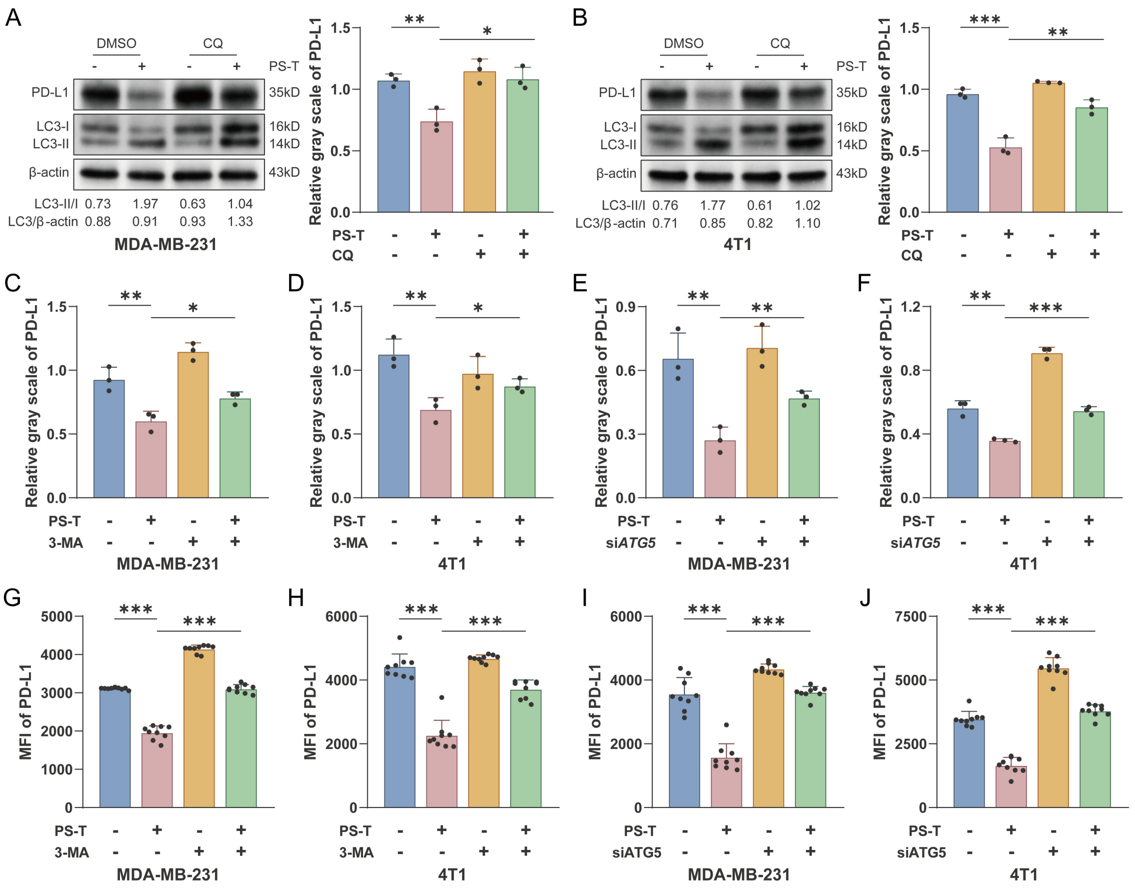

Fig. S13. Autophagy inhibition reverses the PS-T–induced degradation of PD-L1

(A-B) The relative gray density of PD-L1 in MDA-MB-231 (A) and 4T1 cells (B) that autophagy was inhibited by CQ (n=3). **(C-F)** The relative gray density of PD-L1 in MDA-MB-231 and 4T1 cells that autophagy was inhibited by 3-MA (C-D) and si*ATG5* (E-F) (n=3). **(G-J)** The mean fluorescence intensity (MFI) of PD-L1 protein in MDA-MB-231 and 4T1 cells that autophagy was inhibited by 3-MA (G-H) and si*ATG5* (I-J) (n=9).

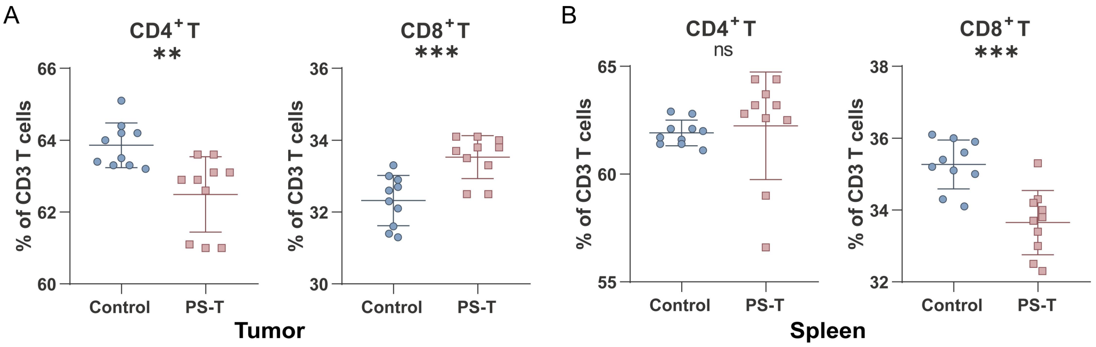

Fig. S14. Percentage of CD4⁺ T and CD8⁺ T cells in T lymphocytes

(A-B) The percentage of CD4⁺ T (*left*) and CD8⁺ T cells (*right*) in tumor (A) and splenic (B) infiltrating T lymphocytes (n=10). The data were combined based on two independent experiments. (mean ± standard deviation; ns, not significant; $^{**}P < 0.01$; $^{***}P < 0.001$).

Supplementary Tables

Table. S1. Clinicopathological characteristics of TNBC patients

No.	Age (years)	Tumor Size (cm)	Lymph Node Status	Histological Grade	Ki67 Index (%)	LC3 Expression Level	PD-L1 Expression Level
1	57	1.0	Positive	I	10	High	Low
2	75	5.0	Positive	II	40	High	Low
3	50	3.0	Negative	II	90	High	Low
4	68	3.0	Positive	II	80	High	Low
5	39	3.0	Positive	I	20	High	Low
6	41	2.0	Negative	II	40	High	Low
7	39	3.0	Negative	II	8	High	Low
8	50	3.0	Positive	II	30	High	Low
9	43	4.0	Positive	II	30	High	Low
10	55	5.0	Negative	II	30	High	Low
11	28	4.0	Positive	II	80	Low	High
12	71	2.5	Negative	II	30	Low	High
13	78	4.5	Negative	II	20	Low	High
14	68	3.0	Negative	II	50	Low	High
15	51	3.0	Positive	II	40	Low	High
16	57	4.0	Negative	II	40	Low	High
17	61	2.0	Negative	I	30	Low	High
18	66	2.5	Positive	II	15	Low	High
19	47	3.0	Positive	II	15	Low	High
20	39	2.8	Negative	II	50	Low	High

Table. S2. The TPM of *CD274* mRNA levels in the tumor infiltrating immune cells

	Control	PS-T	*P* value
**Myeloid**	0.24	0.64	0.08
**MDSC**	0.11	0.23	0.41
**DC**	0.30	0.60	0.15
